# Real-World Management and Patterns of Antibiotic Use in Patients with Keratitis and Ocular Surface Injuries: A Nationwide Survey Study in Spain

**DOI:** 10.3390/antibiotics15090856

**Published:** 2026-09-01

**Authors:** Francisco Arnalich-Montiel, David J. Galarreta, María Nieves Díez García

**Affiliations:** 1 Cornea Unit, Department of Ophthalmology, Hospital Universitario Ramón y Cajal, Universidad de Alcalá, and Instituto Ramón y Cajal de Investigación Sanitaria (IRYCIS), Carretera de Colmenar Viejo km 9.1, 28034 Madrid, Spain; 2 University Center for Health Sciences-HM Hospitals (CUHMED), Camilo Jose Cela University, 28692 Madrid, Spain; 3 Spanish Network of Inflammatory Diseases and Immunopathology of Organs and Systems (REI-RICORS; RD24/0007/0020), Institute of Health Carlos III, Ministry of Science and Innovation, 28029 Madrid, Spain; 4 Hospital Clínico Universitario de Valladolid and Instituto Oftalmológico Recoletas, C/General Ruiz 4, 47004 Valladolid, Spain; dgalarreta@saludcastillayleon.es; 5 Spanish Network of Inflammatory Diseases and Immunopathology of Organs and Systems (REI-RICORS; RD24/0007/0004), Institute of Health Carlos III, Ministry of Science and Innovation, 28029 Madrid, Spain; 6 Medical Department, Esteve Pharmaceuticals, S.A., 08038 Barcelona, Spain; mdiez@esteve.com

**Keywords:** keratitis, ocular trauma, ocular surface, bacterial keratitis, viral keratitis, fungal keratitis

## Abstract

**Background/Objectives**: Infectious keratitis and eye injuries, despite being well-recognized ophthalmological diseases, continue to pose diagnostic and therapeutic challenges in clinical practice; therefore, this study was conducted to assess the salient features of care provided by Spanish ophthalmologists working in emergency and ocular surface consultations to patients presenting with keratitis and ocular trauma. **Methods**: Nationwide cross-sectional survey with the participation of 100 ophthalmologists, including 50 ophthalmologists in emergency care and 50 ocular surface specialists. The study questionnaire assessed diagnostic approaches, treatment strategies, and factors influencing therapeutic decision making. **Results**: The mean age of participants (51% women) was 37.9 years, with 10.2 years of professional activity. External eye examination, slit lamp examination, fluorescein staining, computed tomography (CT) in penetrating injuries, and microbiological cultures in corneal ulcers were rated as important diagnostic methods. Topical antibiotics were especially recommended for keratitis in general (mean 80.6%) and for contact lens-related keratitis (85.6%). The use of antiseptics was limited. Topical corticosteroids were most commonly selected for viral keratitis with stromal and endothelial involvement (between 24.3% and 50.4%); chlorhexidine (52.0%) and propamidine (26.8%) for Acanthamoeba keratitis; re-epithelizing compounds (20.4%) and artificial tears (29.1%) for actinic keratitis; and topical antifungals for fungal keratitis (77%). **Conclusions**: This study highlights a limited availability of some specific diagnostic methods and an extensive use of topical antibiotics in the management of keratitis and ocular surface injuries, with relatively limited use of antiseptics. Areas of improvement in therapeutic approach would be awareness of a potential overuse of topical antibiotics with the consequences of increase in antimicrobial resistance.

## 1. Introduction

Keratitis, corneal ulcers, and eye injuries constitute an important group of ophthalmological disorders that impair vision in millions of people worldwide [1]. All of these conditions, if not appropriately treated, may lead to severe complications, including severe visual loss and blindness. Keratitis is associated with both infectious and non-infectious diseases. Infectious keratitis is the most common cause of non-trachomatous corneal opacification and accounted for the fifth cause of moderate/severe visual impairment and blindness in a meta-analysis of population-based data collected from the Global Vision Database from 1980 to 2014 [2]. A report of the World Health Organization in 2002 stated that 5.1% of blindness was estimated to be a result of corneal opacification [3].

Infectious keratitis can be caused by a wide range of microorganisms, including bacteria, fungi, virus, parasites, and polymicrobial infection [4,5]. Viral, Acanthamoeba keratitis, and mycotic keratitis especially caused by uncommon fungal species are challenging to diagnose and treat [6,7,8]. Contact lens wear, trauma, underlying ocular surface diseases, lid diseases, post-ocular surgery, topical steroid use, and systemic immunosuppression are well-known risk factors for keratitis [9]. On the other hand, ocular injuries from traumatic accidents, exposure to chemicals or foreign objects due to blunt contusions or penetrating injuries occurring at work and home environments are significant factors of serious complications and visual loss [10,11]. Ocular injuries are common ophthalmological emergencies that require detailed history and assessment of ocular structures to determine whether patients should be referred to specialists for further investigation and eventual surgical repair [12,13].

Timely treatment of keratitis and eye injuries are crucial for preventing blindness. The initial treatment of infectious keratitis typically involves high-dose and high-frequency administration of broad-spectrum antibiotics in eye drops or ointments. However, inadequate drug delivery due to ocular surface barrier, low bioavailability, ocular surface toxicity risks, emergence of drug resistance, including multidrug-resistant bacteria strains, and presence of biofilms pose considerable obstacles in achieving effective therapeutic outcomes [14]. Innovative treatment methods (e.g., nanoparticles, nanogels, nanoemulsions, quantum dots, hydrogels, etc.) and novel therapies (e.g., plasma therapy, phage therapy, gene therapy, and corneal cross-linking) offer new insights into keratitis treatment strategies [15,16].

However, infectious keratitis and eye injuries, despite being well-recognized ophthalmological diseases, continue to pose diagnostic and therapeutic challenges due to geographical disparities and limited comprehensive nation-level data on clinical characteristics and treatment approach. Therefore, assessment of practice patterns in the treatment of these conditions is essential for guiding public health interventions and improving patient outcomes [17,18]. The purpose of this real-world study was to characterize the current management of keratitis and eye injuries based on a survey of ophthalmologists and to identify areas of improvement. A distinctive feature of the study was the description of clinical practice patterns among ophthalmologists working in the emergency setting and those working in ocular surface consultations.

## 2. Results

### 2.1. Characteristics of Participants

A total of 100 ophthalmologists (51% women) with a mean (± SD) age of 37.9 ± 9.1 years (range 26–64 years) and mean years of professional activity of 10.2 ± 8.9 years participated in the study providing insights into the diagnostic and therapeutic management of keratitis and ocular surface injuries in both emergency and specialized clinical settings. Participants included 50 professionals in the emergency ophthalmology group and 50 in the cornea and ocular surface consultations group, with a similar distribution by age, sex, and years of practice. A total of 52% of participants worked in a public center exclusively and 41% in both public and private centers, with level III hospitals recorded in 73% of cases. In Spain, ophthalmologists in the emergency setting also work in other ophthalmology units in addition to attending emergency cases.

In the emergency setting, ophthalmologists reported a mean of 359.9 ± 201.1 monthly visits, including 90.1 ± 78.3 new patients with ocular trauma/surface lesion, and 60.7 ± 63.1 patients under active treatment. The corresponding figures for specialists in ocular surface were 346.6 ± 203.5, 80.8 ± 71.5, and 54.9 ± 45.1, respectively. The distribution of ocular pathologies among ophthalmologists from the emergency setting and ocular surface specialists is shown in Table 1.

### 2.2. Patients with Keratitis/Corneal Ulcers: Diagnosis and Clinical Pathway

In patients with keratitis/corneal ulcers, ophthalmologists reported that the percentages of men (48.1%) and women (51.9%) were similar, and the age range 40–60 years and over 60 years accounted for 57.3% of patients. In the emergency ophthalmology setting, the diagnostic methods considered the most important, those with a mean score above 9, were external eye examination, slit lamp examination, fluorescein staining, and computed tomography (CT) in penetrating injuries (Table 2). Ocular surface specialists coincided with higher scores for external eye examination and slit lamp examination for patients with keratitis and corneal ulcers, as well as eyelid/eyelashes examination and microbiological cultures in corneal ulcers (Table 2).

The types of keratitis that ophthalmologists considered easiest to diagnose and differentiate were exposure keratopathy (mean score 9.1) and allergic keratoconjunctivitis (8.9), followed by neurotrophic keratitis (7.8) and bacterial keratitis (7.7). Fungal (mean score 4.7) and protozoal keratitis (3.7) were the most difficult to diagnose. Also, 94% of specialists considered that an adequate diagnosis of keratitis was being performed at their center. Establishment of specialized ocular surface and cornea units with trained and engaged professionals was cited as a major advance for improving the diagnosis of keratitis.

**Figure 1:**
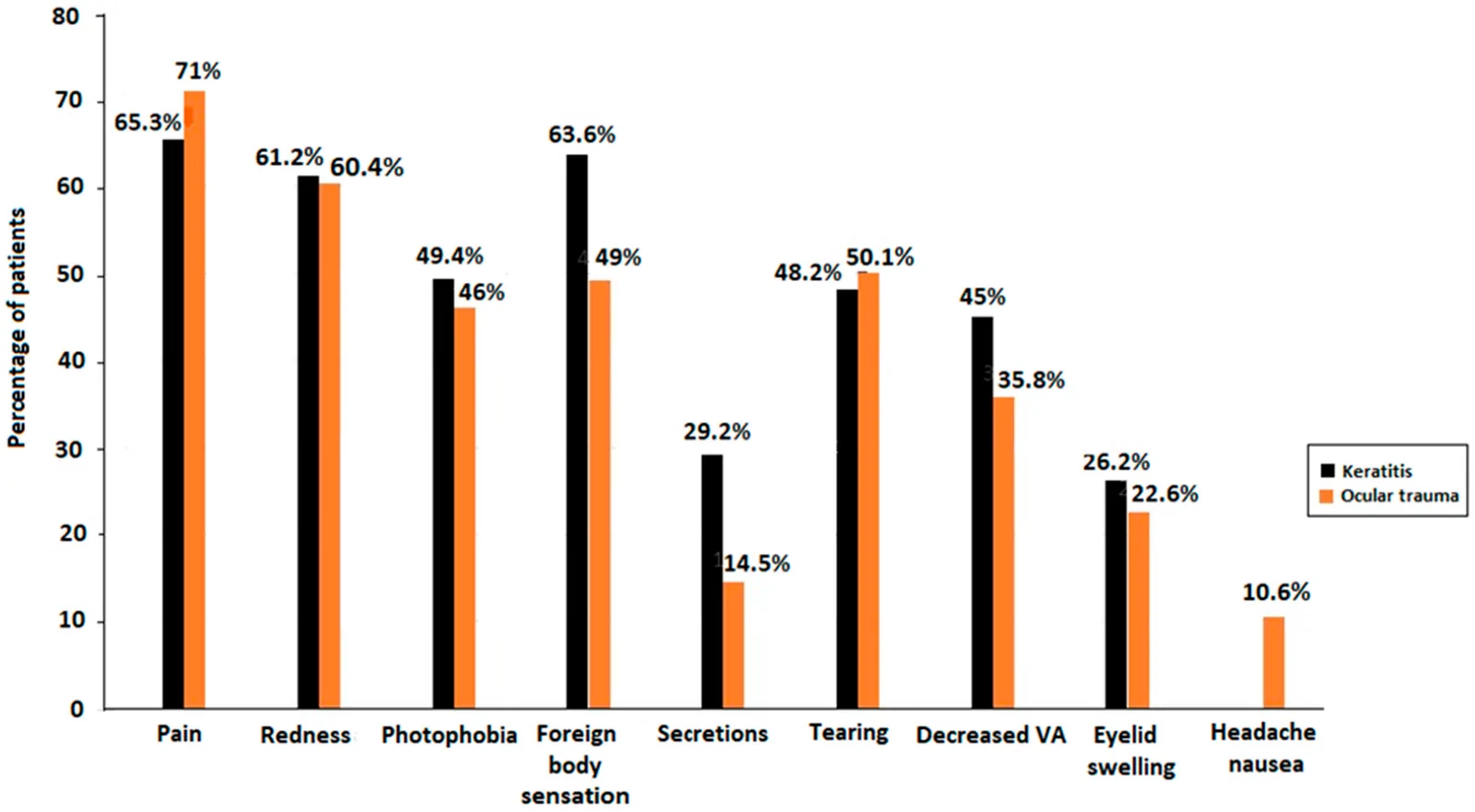
Mean percentage of symptoms reported by patients with keratitis and ocular trauma/ocular surface lesion before entering the clinical care pathway in the emergency setting (VA: visual acuity). Multiple symptoms could be selected.

According to the experience of the ophthalmologists, pain, foreign body sensation, and redness present in 65.3%, 63.6% and 61.2% of patients, respectively, were the most common symptoms before entering the clinical care pathway (Figure 1). Access to the healthcare system in the emergency setting included the patient’s own initiative in 54.6% of cases or via referral by primary care physicians in 21.1%, other ophthalmologists in 4.9%, opticians in 4.3%, and community pharmacists in 2.4%. In ocular surface consultations, patients were referred from the emergency setting in 51.3% of cases, by other ophthalmologists in 14.2%, primary care physicians in 13.5%, opticians in 3.7%, and community pharmacists in 1.2%.

Almost all respondents diagnosed and treated patients (99%), while the large majority (92%) also conducted follow-up. In the case of referrals, particularly in patients with poor clinical course and inadequate response to treatment, 75% of ophthalmologists referred patients to a specialized anterior eye/cornea unit. Also, protozoal and fungal keratitis were the types of keratitis followed for the longest periods, with a mean of 11 and 8.9 follow-up visits, respectively, whereas actinic keratitis was scheduled or an average of only one follow-up visit.

### 2.3. Treatment of Keratitis

The main criteria for starting treatment with antibiotics in bacterial keratitis were infection or suspicion of infection reported by 37.4% of participants, corneal infiltration by 23.2% and abundant exudation (yellow or green discharge) by 13.1%. In patients with actinic keratitis, antibiotics were indicated in the presence of epithelial defects of >1 mm, suspicion of bacterial superinfection or prophylactically. In patients with viral keratitis, the main criteria for treatment with antibiotics were superinfection or risk of superinfection (43.2%) and large fluorescein-positive epithelial defect (24.7%). The main criteria for treating fungal keratitis with antifungals were clinical suspicion (31%) and a microbiologically confirmed diagnosis (30%).

As shown in Table 3 and Table 4, treatment of patients with keratitis using monotherapy varied according to the type of keratitis, but some modality of topical treatment was selected in all cases. Topical antibiotics were especially recommended for keratitis in general (mean 80.6%) and keratitis due to contact lens wear (85.6%). In actinic keratitis, topical antibiotics were recorded in a mean of 26.9% of cases. Topical corticosteroids were most commonly selected for viral keratitis with stromal and endothelial involvement (between 24.3% and 50.4%); polyhexanide (PHMB) (52%) and hexamidine (26.8%) for Acanthamoeba keratitis; re-epithelizing compounds (20.4%) and artificial tears (29.1%) for actinic keratitis; and topical antifungals for fungal keratitis (77%). In fungal keratitis, topical antibiotics were administered in a mean of 4.7% of cases. Systemic antivirals and antifungals were mostly limited to patients with viral or fungal keratitis.

Treatment of keratitis using combined therapy included a variety of different combinations of topical and systemic agents. Table 5 shows the two most frequently prescribed combined treatment for each type of keratitis. The most common combinations were topical antibiotics + topical antiseptics (mean frequency of 39.3%) and topical antibiotics + topical corticosteroids (38.6%) for bacterial keratitis; topical antibiotics + lid wipes + re-epithelizing ointment + artificial tears (68.7%) for contact lens-related bacterial keratitis; chlorhexidine eye drops + propamidine eye drops (57.7%) and chlorhexidine eye drops + topical antibiotics + topical corticosteroids (56.5%) for Acanthamoeba keratitis; and topical antibiotics + re-epithelizing ointment + artificial tears + cycloplegic (52.8%) for actinic keratitis.

Topical antibiotics + topical antivirals + topical corticosteroids associated with systemic antivirals were the most frequent combinations in the different types of viral keratitis (Table 4). Fungal keratitis was mostly treated with topical antifungals + antiseptics (44.6%) and topical antibiotics + topical antifungals + topical corticosteroids (41.2%).

In relation to the importance of treatment-related characteristics considered by participants when starting treatment for keratitis, the highest mean scores were obtained for antibacterial, antiviral, and antifungal efficacy (mean 9.7), favorable evidence from clinical practice/real-world data (8.9), supportive high-quality evidence (8.8), and usefulness in reducing and achieving closure of the ulcer without causing haze (8.6). Moreover, treatment cost was considered an important barrier, with a mean score of 6.6, particularly in patients with non-infectious intercurrent corneal ulcers associated with dermatological, rheumatological, or neurological diseases (mean score 7.0).

### 2.4. Management of Ocular Trauma/Ocular Surface Lesion in the Emergency Setting

The most frequent symptoms experienced by patients with ocular trauma/ocular surface lesion prior to entering the clinical care pathway were quite similar to those reported for patients with keratitis (Figure 1). Pain and redness were present in more than 60% of patients. Most patients (63%) accessed the system on their own initiative. Almost all participants diagnosed and treated patients (98%), while the large majority (78%) also conducted follow-up. Most participants (89%) referred patients to the cornea and ocular surface subspecialty, especially in severe conditions or complications (e.g., perforation, deep foreign bodies, or fracture of the orbital floor). Patients with penetrating trauma were followed for the longest period (average of eight follow-up visits), whereas those with foreign body were scheduled for about one follow-up visit. The mean frequency of visits was 2.1 days for penetrating injuries and 6.9 days for blunt trauma.

Therapeutic options for patients treated with monotherapy are shown in Table 6. Topical antibiotics were recommended in a mean of 55.2% of cases of ocular trauma/ocular surface lesions and antiseptics in 10.9%. In relation to the type of ocular trauma, topical antibiotics were given in 72.9% of cases of ocular trauma with foreign body, 44.1% in burns due to chemical products, and in 33% in penetrating injuries. Antiseptics were recommended in a mean of 16.9% of cases with foreign body, 11.5% of burns due to chemical products, and 1.1% of penetrating injuries. Topical corticosteroids were especially considered in burns due to chemical agents (40.8%) and systemic antibiotics in penetrating injuries (61.1%). Moreover, topical antibiotics were also present in all combined therapies, followed by re-epithelizing ointment (Table 7). Systemic antibiotics were indicated in penetrating injuries.

In addition, the most valuable characteristics of treatment options included antibacterial, antiviral, and antifungal efficacy (mean 9.3), high level of evidence (9.0), and usefulness for closing the injury without haze (8.7). The importance of cost of treatment as a therapeutic barrier was moderate (mean 6.7).

## 3. Discussion

This survey study carried out among ophthalmologists provides an overview of the profile of patients with keratitis and ocular trauma attended in daily practice, particularly in relation to clinical care pathways, diagnostic aspects and therapeutic management. The assessment of characteristics of different types of keratitis and ocular trauma with ocular surface lesions is a distinctive feature of the study as compared to previous publications based on practice patterns surveys among specialists of specific conditions, such as empirical treatment of bacterial ulcers [17], management of bacterial keratitis [19,20], open globe injury [21], or traumatic corneal abrasion [22]. Moreover, the distinction between opinions provided by ophthalmologists working in the emergency department and those working in cornea and ocular surface consultations is a further contribution of the study.

Participants were in the middle-age group, had a mean of 10 years of clinical experience, and the majority worked in tertiary care centers distributed throughout the Spanish territory. Ophthalmologists in the emergency care setting primarily attended patients with non-infectious keratitis and traumatic ocular lesions, whereas ocular surface specialists mainly managed patients with bacterial keratitis, including Acanthamoeba keratitis, and other less common viral and fungal corneal infections. All participants in both healthcare settings had a high workload according to the number of patients with ocular surface lesions attended on a monthly basis.

In the diagnostic approach, all participants independently of their working place assigned a high score of importance for careful and complete external and slit lamp examination to assess the morphological features of lesions, together with fluorescein staining in keratitis, computed tomography (CT) imaging in open ocular trauma, microbiological culture of corneal scrapings as needed, and vital staining especially recommended by ocular surface specialists in both keratitis and ocular trauma. Other techniques, such as assessment of corneal sensitivity, confocal microscopy, serological testing, microbiological culture, and PCR for rapid microbial identification were rated less important in their application in routine practice, indicating limited use of diagnostic methods.

Treatment of keratitis and eye injuries showed a diversity of therapeutic modalities, particularly regarding the use of combined therapy. Overall, one of the main findings of the study was a high reliance on topical antibiotics across different scenarios, which were used as monotherapy or in combination with other agents. The present results may reflect a tendency toward broad antibiotic prophylactic prescribing in routine clinical practice.

In infectious keratitis, broad-spectrum antibiotics remain the primary recommended treatment, with therapeutic choices depending on results obtained by smears and local antimicrobial resistance patterns. In bacterial keratitis, the mean percentage of use of topical antibiotics in monotherapy was 80.6%, which is consistent with evidence based on systematic reviews and clinical practice guidelines for the recommendation of topical antibiotic eye drops as the preferred method for the initial treatment of bacterial keratitis [23,24]. In around 38.6% of cases, topical antibiotics were combined with corticosteroids. The role of corticosteroids as adjunctive therapy for bacterial keratitis has not been clearly defined, and optimal use requires appropriate timing and careful dose regulation with the minimum amount to control inflammation. The Steroids for Corneal Ulcers Trial (SCUT) assessed the effect of adjunctive topical corticosteroids on clinical outcomes in patients with bacterial corneal ulcers, and found no differences in 3-month spectacle-corrected visual acuity (SBCVA) (primary endpoint) as compared with placebo. However, corticosteroid treatment was associated with a benefit in visual acuity compared with the placebo group in the subgroups with the worst visual acuity and central ulcer location at baseline. No safety concerns were registered [25]. Moreover, in a prospective subanalysis of SCUT focused on Nocardia keratitis with data at 3 and 12 months, the use of adjunct corticosteroids resulted in larger infiltrate/scar sizes, suggesting they may not be appropriate for treating this disease [26,27]. It was also found that patients with non-Nocardia keratitis given topical corticosteroids early in the course of infection within 2 to 3 days of antibiotic therapy had approximately 1-line better visual acuity at 3 months that did those given placebo [28]. These nuances are clinically important when interpreting the 38.6% rate of combined antibiotic–corticosteroid use observed in the present survey, as inappropriate use in certain bacterial etiologies could lead to worse outcomes. However, much of the literature shows no difference in clinical outcome with the addition of corticosteroids [24].

The use of topical antibiotics was also recorded in a high percentage of infectious keratitis in contact lens wearers (85.6%), but mean percentages of use decreased to 26.9% in actinic keratitis and less than 10% in viral and fungal keratitis. Antiviral agents were used as primary treatment in 71.2% of cases of HSV epithelial keratitis, decreasing to 12.6% and 8.1% in HSV stromal and endothelial keratitis. Topical antibiotics in monotherapy showed a limited use in viral keratitis (HSV 2.4% to 7.6%; VZV 0.9% to 8.1%; epidemic adenoviral keratitis 3.8% to 8.2%). In combination therapies, however, 67.4% of participants added topical antibiotics to topical antivirals in HSV epithelial keratitis and up to 61.8% in VZV epithelial keratitis. Furthermore, combination regimens including topical antibiotics were also reported for stromal involvement: 10 participants added them alongside systemic antivirals and topical corticoids in 41.5% of HSV stromal keratitis cases, while a similar combination was used by 10 participants in 33.6% of VZV stromal keratitis cases. Antivirals alone are the treatment of choice for HSV epithelial keratitis [29] and uncomplicated VZV epithelial keratitis [30]. For HSV and VZV stromal and epithelial keratitis, topical corticosteroids combined with topical and systemic antivirals was the treatment more commonly selected, which is consistent with guidelines recommendations [29,31].

There was also a remarkable use of topical antibiotics in the emergency setting among patients with ocular trauma (55.2%), particularly in eye injuries with foreign body (72.9%), for burns due to chemical products the use of topical corticosteroids (40.8%) was similar to that of antibiotics (44.1%). In corneal abrasion, antibiotic prophylaxis in preventing ocular infection or accelerating epithelial healing is of very low certainty according to the analysis of 998 patients included in a recent systematic review and meta-analysis of randomized controlled trials [32]. In contrast, the use of topical steroids in the acute management of chemical burns is widely accepted [33], which is consistent with our findings.

While topical antibiotics are recommended as empirical or culture-directed treatment for bacterial keratitis, their use in non-infectious or prophylactic contexts (e.g., corneal abrasion, ocular trauma, and viral keratitis) represents a practice that may substantially contribute to antibiotic overuse. Overuse of topical antibiotics has been recognized as an important driver in antimicrobial resistance (AMR), an emerging concern with significant clinical implications [34]. A recent in-depth review of AMR in bacterial keratitis reported pooled data from studies conducted in Australia, Asia, North America, and the UK, showed variable resistance rates, with up to 35% resistance to cephalosporins, 47% to fluoroquinolones, and 52% for aminoglycosides in Gram-positive infections, and up to 60%, 57%, and 50% resistance rates to these antibiotics in Gram-negative infections [35]. Thus, the effectiveness of broad-spectrum topical antibiotics is being challenged by AMR, including multidrug resistance, and treatment costs [4,36].

In response to the growing problem of AMR, ophthalmic practices have increasingly shifted away from antibiotic-based prophylaxis toward antiseptic alternatives. Antiseptics have demonstrated high efficacy in reducing microbial load without contributing to antibiotic resistance. Some antiseptics, such as povidone-iodine (PVP-I) present broad-spectrum antimicrobial activity and can be also used in the treatment of ocular surface infections, including adenovirus keratoconjunctivitis and bacterial keratitis [37]. In our study, however, the mean percentage of use of antiseptics in bacterial keratitis was reduced to 8.7%. The use of antiseptics in patients with keratitis has been evaluated in a few studies. In one study, it was shown that chlorhexidine proved effective for fungal corneal ulcers [38] and new better tolerated antiseptics like ozonated oil in liposomes seem to aid in the management of keratitis and corneal ulcers [39,40], another eye drops formulation with antiseptic and re-epithelizing properties was effective in patients with punctate keratitis [41].

The patterns of treatment for patients with different types of keratitis, such as polyhexanide (PHMB) and hexamidine in Acanthamoeba keratitis, antivirals and topical corticosteroids in viral keratitis, and topical and systemic antifungal agents in fungal keratitis are consistent with evidence reported in the literature [42,43]. In the management of Acanthamoeba keratitis, topical chlorhexidine (52.0%) and propamidine (Brolene^®^) (26.8%) were the most frequently recommended options, which are in line with drug treatment guidelines for this rare form of keratitis [44]. A recent systematic review and meta-analysis of the efficacy and safety of PHMB for Acanthamoeba keratitis based on 17 studies and 923 eyes showed a pooled proportion of cure of 75.7%, with the highest cure rate with PHMB 0.08% monotherapy, supporting its use as a first-line empirical treatment [45]. In our study, PHMB was used in only 13.3% of cases. However, it should be noted that solid evidence based on systematic reviews and meta-analyses assessing the effectiveness of topical antibiotics versus antiseptics in infectious and non-infectious keratitis is lacking.

Data on specific antiviral and antifungal drugs, doses, and treatment regimens were not evaluated, although antibacterial, antiviral, and antifungal efficacy, as well as a high level of evidence were rated as very important factors in the clinician’s therapeutic choices. Also, opinions regarding the schedule of follow-up visits were consistent with the severity of diseases, with longer follow-up periods and shorter visit intervals in penetrating injuries and protozoal and fungal keratitis.

It has been shown that bacterial keratitis represents a significant economic burden on patients and healthcare systems [46,47], and in this respect, our study also showed that cost of treatment was rated as an important barrier in the management of keratitis especially in patients with non-infectious corneal ulcers and comorbidities. Another interesting aspect of the study is the fact that ophthalmologists reported to take integral care of patients, including diagnosis, treatment, and follow-up, and to refer patients to specialized cornea and ocular surface units in cases of torpid evolution and inadequate response to treatment.

The present findings should be interpreted considering the limitations of survey studies, particularly its descriptive design, data based on self-reported practices, selection bias due to voluntary participation, and the fact that response/completion rates were not assessed. Moreover, the study questionnaire was developed by two specialists based on a literature review and clinical experience, but neither pilot testing nor content validity assessment of the instrument was performed. The age of participants ranged between 26 and 64 years, which may affect the generalizability of results, but also reflects the real-world characteristics of the study. However, the assessment of opinions regarding the diagnostic and therapeutic aspects of keratitis and ocular trauma from actively practicing ophthalmologists working in emergency departments and specialized ocular surface units is a strength of the study.

## 4. Materials and Methods

### 4.1. Study Design and Participants

This was a nationwide cross-sectional survey study (The IMPAQTO Study, Spanish acronym for EpIdemiología y Manejo de PAcientes con Queratitis y Traumatismo Ocular en España, Epidemiology and Management of Patients with Keratitis and Ocular Injury in Spain). The Checklist for Reporting Of Survey Studies (CROSS) was followed [48] (Table S2, Supplementary Materials). The primary objective of the study was to assess the workload represented by keratitis and ocular surface injuries in ophthalmology emergency and ocular surface consultation settings in Spain. The secondary objective was to evaluate how patients with keratitis and ocular surface injuries are managed in both settings. Inclusion criteria were as follows: (a) specialists in emergency ophthalmology or third- and fourth-year ophthalmology residents who provided clinical care in the emergency department at least 2 days per week, and (b) ocular surface specialists who managed patients with corneal diseases and ocular surface injuries in their daily practice. Exclusion criteria were specialists with less than 2 years of experience (i.e., first- and second-year residents) and those over 65 years of age in either emergency ophthalmology or ocular surface specialties.

As no patient-level data were collected and participation was anonymous, formal ethics committee approval was not required according to local regulations. To safeguard privacy, study data were processed in accordance with Spanish Data Protection Law 3/2018 using a de-identified database to ensure full confidentiality. All participants provided electronic informed consent before completing the questionnaire.

### 4.2. Study Procedures

The study questionnaire was developed by two experienced ophthalmology specialists in corneal and anterior segment diseases (F.A.-M. and D.G.M.), who discussed and developed the study questionnaire based on an extensive review of the literature and their own clinical expertise. These specialists who formed the scientific committee were also responsible for supervision of the progression of the study, including the analysis and interpretation of data.

The final questionnaire included nine sections with items and subitems covering the following aspects: (a) demographic and professional data of participants (4 items); (b) data of the centers (6 items); (c) profile of patients with ocular surface lesions (keratitis/corneal ulcer (10 items); (d) clinical care pathway of patients with keratitis/corneal ulcer (3 items); (e) treatment of the different types of keratitis (13 items); (f) drivers in the treatment of keratitis (4 items); (g) clinical care pathway of patients with ocular trauma/ocular surface lesion from the emergency department (3 items); (h) treatment of ocular trauma/ocular surface lesions (8 items); and (i) drivers in the management of ocular trauma/ocular surface lesions (4 items).

Also, some of the sections included separate responses for subspecialties in emergency ophthalmology and specialists in ocular surface diseases. The questions included open-ended responses, multiple-choice options, or categorization of answers on a scale from 0 to 10 points, where 0 was not important at all and 10 was very important. The study questionnaire is described in Table S1 (Supplementary Materials).

Participants were ophthalmologists practicing in Spain included in the database of Esteve, a Spanish international pharmaceutical company headquartered in Barcelona with more than 90 years of experience. Participants were recruited through invitations distributed via e-mail that included a brochure with full information on the project. Participation in the study was anonymous and confidential. No personal identifying information was collected and all responses were analyzed in aggregate form. The study questionnaire was lodged in an Internet microsite to which participants accessed via a weblink included in the e-mail. Physicians who accepted to participate in the study were provided with the microsite URL and the user’s password. Data were collected over a 3-month period from 3 February to 5 May 2025.

### 4.3. Statistical Analysis

According to the objective of the study, which was to assess the management of keratitis and ocular surface injuries in ophthalmology emergency departments and ocular surface consultations, a sample size of 100 participants was required, assuming a margin of error of approximately 10%, a 95% confidence interval (CI), and 50% heterogeneity. The 100 participants were divided into specialists in emergency ophthalmology (*n* = 50) and ocular surface ophthalmology specialists (*n* = 50). Only fully completed questionnaires were analyzed. Recruitment was closed once the target sample size had been reached. Categorical data are expressed as frequencies and percentages, and continuous data as mean and standard deviation (±SD) and median and interquartile range (IQR) according to the normal or non-normal distribution of data. Data were analyzed using the SAS statistical software (version 9.1.3) (Statistical Analysis Systems, SAS Institute, Cary, NC, USA).

## 5. Conclusions

This survey study, carried out among ophthalmologists working in ophthalmology emergency and ocular surface consultation settings, provides insights into the clinical pathway and diagnostic and treatment practices for patients presenting with corneal infection and eye injuries. Relevant findings included the high workload of participants, the generally limited availability of some specific diagnostic methods, and the extensive use of topical antibiotics, probably to the detriment of the use of antiseptics. The present data offer valuable information for the management of patients with keratitis and ocular trauma, particularly focused on areas of improvement in the diagnostic process and therapeutic approach. This would help in preventing the potential overuse of topical antibiotics, particularly in non-infectious/prophylactic contexts, as well as the associated increase in antimicrobial resistance and treatment cost.

## Figures and Tables

**Table 1:** Differences between emergency setting ophthalmologists and ocular surface specialists in the distribution of presenting disorders.

Ophthalmologists from the Emergency Setting		Ocular Surface Specialists	
Non-infectious keratitis	19.2%	Non-infectious keratitis	36.1%
Ocular trauma with ocular surface lesion	18.6%	Viral keratitis	17.9%
Foreign body	18.1%	Contact lens-related keratitis	15.9%
Non-infectious corneal ulcers	9.8%	Non-infectious corneal ulcers	11.4%
Infectious keratitis/corneal ulcers	8.8%	Fungal keratitis	1.8%
Blunt trauma	7.6%	Amoebic keratitis	1.3%
Chemical agents-related burns	4.3%		
Actinic keratitis	3.6%		
Penetrating injuries	1.3%		

**Table 2:** Importance assigned by ophthalmologists to different methods for the diagnosis of ocular surface lesions.

Diagnostic Methods	Emergency Ophthalmologists (*n* = 50)		Ocular Surface Specialists (*n* = 50)	
	Ocular Trauma/Ocular Surface Lesion Mean ± SD	Keratitis/ Corneal Ulcer Mean ± SD	Keratitis Mean ± SD	Corneal Ulcer Mean ± SD
External eye exam, visual acuity (and pain severity)	9.2 ± 1.2	8.4 ± 1.9	9.3 ± 1.3	9.2 ± 1.6
Slit lamp: location, shape, appearance, size, margins, infiltration	9.9 ± 0.3	9.9 ± 0.2	9.4 ± 1.7	9.8 ± 0.8
Eyelid and eyelashes (eyelid eversion)	8.5 ± 2.1	8.2 ± 2.3	NA	NA
Fluorescein staining	9.8 ± 0.7	9.8 ± 0.5	NA	NA
Corneal sensitivity	4.3 ± 3.0	6.8 ± 2.7	5.7 ± 3.1	7.2 ± 2.8
IOP measurement, ophthalmoscopy	8.1 ± 2.1	5.7 ± 2.7	NA	NA
CT in penetrating open lesions, orbital fracture	9.5 ± 1.1	4.5 ± 4.2	NA	NA
Microbiological examination after corneal scraping: Gram, calcofluor white, acid-fast staining, etc.	5.1 ± 3.4	8.9 ± 1.4	3.6 ± 3.0	7.9 ± 2.9
Culture blood agar, chocolate agar, Sabouraud, thioglycolate broth	NA	NA	4.1 ± 3.4	9.0 ± 1.8
Microbiological examination after corneal scraping: PCR	3.9 ± 3.5	6.9 ± 2.6	4.1 ± 3.5	7.4 ± 2.6
Visual field examination, papillary response, optic nerve function	7.0 ± 3.1	2.9 ± 2.8	NA	NA
Confocal microscopy	1.7 ± 2.0	4.1 ± 3.2	2.4 ± 2.7	3.8 ± 3.3
B-mode echography	6.3 ± 2.9	2.7 ± 2.3	NA	NA
Systemic studies: blood tests, urinalysis, renal function, serological tests: ANA, ANCA, FT-ABS, etc.	NA	NA	4.5 ± 3.1	4.4 ± 2.7
Vital staining: fluor, lissamine green	NA	NA	8.5 ± 2.7	8.3 ± 2.8

SD: standard deviation; IOP: intraocular pressure; CT: computed tomography; PCR: polymerase chain reaction; ANA: antinuclear antibodies; ANCA: anti-neutrophil cytoplasmic antibodies; FT-ABS: Fluorescent treponemal antibody absorption test; NA: not applicable.

**Table 3:** Monotherapy treatment in patients with bacterial keratitis.

Therapeutic Agents	Bacterial Keratitis				Acanthamoeba Keratitis		Actinic Keratitis	
	All Cases		Contact Lens Wear					
	% Mean ± SD	% Median (IQR)	% Mean ± SD	% Median (IQR)	% Mean ± SD	% Median (IQR)	% Mean ± SD	% Median (IQR)
Topical treatment								
Antibiotics	80.6 ± 21.9	80 (70–100)	85.6 ± 22.2	100 (78.7–100)	2.1 ± 5.1	0 (0–0)	26.9 ± 28.5	20 (0–50)
Antiseptics	8.7 ± 11.8	15 (5–50)	4.3 ± 8.9	0 (0–5)			5.1 ± 11.4	0 (0–5)
Lid wipes	3.1 ± 6.4	5 (0–40)	1.1 ± 3.9	0 (0–0)				
Corticosteroids					1.0 ± 3.1	0 (0–0)	13.5 ± 24.9	0 (0–10)
Re-epithelizing ointment			2.3 ± 5.5	0 (0–0)			20.4 ± 22.0	11 (0–30)
Artificial tears			6.0 ± 14.5	0 (0–0)			29.1 ± 26.8	20 (10–43.7)
Chlorhexidine					52.0 ± 34.11	50 (25–80)		
Polyhexanide (PHMB)					13.3 ± 23.0	0 (0–16.5)		
Propamidine (Brolene^®^)					26.8 ± 32.0	15 (0–41.2)		
Hexamidine					3.36 ± 12.9	0 (0–0)		
Cycloplegics							3.4 ± 5.7	0 (0–5)
Systemic treatment								
Antibiotics	2.2 ± 3.8	5 (0–15)	0.7 ± 2.1	0 (0–0)			0.1 ± 0.8	0 (0–0)
Tetracyclines	4.3 ± 6.9	8 (0–38)						
Antifungals					1.4 ± 5.8	0 (0–0)		
Analgesics							1.5 ± 3.8	0 (0–0)

SD: standard deviation; IQR: interquartile range.

**Table 4:** Monotherapy treatment in patients with viral and fungal keratitis.

Therapeutic Agent	Herpes Simplex Virus (HSV) % Mean ± SD			Varicella-Zoster Virus (VZV) % Mean ± SD			Epidemic Adenoviral Keratitis % Mean ± SD			Fungal Keratitis % Mean ± SD
Topical treatment	epithelial	stromal	endothelial	epithelial	stromal	endothelial	epithelial	stromal	endothelial	
Antibiotics	7.6 ± 13.4	3.4 ± 8.7	2.4 ± 7.9	8.1 ± 20.1	1.2 ± 3.7	0.9 ± 3.3	8.2 ± 15.1	5.9 ± 21.2	3.8 ± 16.2	4.7 ± 9.8
Antiseptics	5.2 ± 9.5	2.3 ± 6.6	1.1 ± 3.4	3.0 ± 6.3	1.2 ± 3.7	0.7 ± 2.5	53.2 ± 35.1	21.7 ± 30.4	16.2 ± 27.8	3.4 ± 9.9
Lid wipes	1.3 ± 4.5	0.8 ± 3.1	0.4 ± 1.8	0.8 ± 2.9	0.4 ± 1.9	0.3 ± 1.6	4.8 ± 14.2	3.5 ± 15.4	2.8 ± 14.6	0.7 ± 2.6
Corticosteroids	1.4 ± 4.4	24.3 ± 29.8	29.2 ± 30.1	1.5 ± 5.9	22.9 ± 30.6	24.3 ± 31.0	19.9 ± 25.8	42.2 ± 35.8	50.4 ± 37.1	0.8 ± 0.03
Antivirals	71.2 ± 29.6	12.6 ± 21.4	8.1 ± 18.0	54.9 ± 37.0	14.6 ± 24.2	11.3 ± 23.2	9.4 ± 19.5	7.1 ± 16.4	6.8 ± 15.8	
Cyclosporine							1.9 ± 4.3	10.3 ± 20.9	6.8 ± 15.9	
Antifungals										77.0 ± 26.5
Systemic treatment										
Antibiotics	0.7 ± 3.1	1.0 ± 4.3	0.5 ± 2.8	0.6 ± 3.3	0.5 ± 3.2	0.2 ± 1.2	0.2 ± 1.1	0.4 ± 3.2	1.3 ± 10.2	0.6 ± 3.0
Corticosteroids	0.2 ± 1.2	1.8 ± 10.2	2.5 ± 10.8	0.5 ± 1.9	1.2 ± 3.4	2.4 ± 6.5	0.2 ± 1.1	2.4 ± 11.4	3.2 ± 11.9	0.4 ± 1.4
Antivirals	12.5 ± 21.7	53.7 ± 34.1	56.7 ± 34.6	30.6 ± 34.8	57.9 ± 33.3	59.8 ± 35.0	2.2 ± 10.9	6.4 ± 19.7	8.6 ± 23.6	
Antifungals										12.5 ± 19.9

SD: standard deviation; SDs larger than the mean indicate markedly non-normal distributions for which the mean may misrepresent central tendency.

**Table 5:** Combined therapy in patients with keratitis.

Type of Keratitis		Combined Treatment	Prescription Mean (%)
Bacterial		Topical antibiotics + antiseptics	39.3
		Topical antibiotics + topical corticosteroids	38.6
Contact lens-related bacterial keratitis		Topical antibiotics + lid wipes + re-epithelizing ointment + artificial tears	68.7
		Topical antibiotics + artificial tears	67.0
Acanthamoeba keratitis		Chlorhexidine eye drops + propamidine eye drops	57.7
		Chlorhexidine eye drops + topical antibiotics + topical corticosteroids	56.5
Actinic keratitis		Topical antibiotics + re-epithelizing ointment + artificial tears + cycloplegic	52.8
		Topical antibiotics + topical corticosteroids + artificial tears	43.9
Herpes simplex virus (HSV) keratitis	Epithelial	Topical antibiotics + topical antivirals	67.4
		Topical antivirals + antiseptics	43.1
	Stromal	Systemic antivirals systemic + topical corticosteroids	76.7
		Topical antivirals + systemic antivirals + topical corticosteroids	52.7
	Endothelial	Systemic antivirals + topical corticosteroids	81.6
		Topical antivirals + systemic antivirals + topical corticosteroids	53.2
Varicella-zoster virus (VZV) keratitis	Epithelial	Topical antibiotics + topical antivirals + systemic antivirals	61.8
		Topical antivirals + topical antibiotics	52.4
	Stromal	Systemic antivirals + topical corticosteroids	75.9
		Topical antibiotics + systemic antivirals	47.0
	Endothelial	Systemic antivirals + topical corticosteroids	84.1
		Topical antibiotics + systemic antivirals + topical corticosteroids	53.6
Epidemic adenoviral keratitis	Epithelial	Antiseptics + topical corticosteroids	52.4
		Topical antibiotics + antiseptics + lid wipes	51.7
	Stromal	Antiseptics + topical corticosteroids	71.1
		Topical corticosteroids + cyclosporine	70.2
	Endothelial	Topical corticosteroids + cyclosporine	88.9
		Antiseptics + topical corticosteroids	84.9
Fungal keratitis		Antiseptics + topical antifungals	44.6
		Topical antibiotics + Topical antifungals + topical corticosteroids	41.2

The two more frequent combinations are included for each category.

**Table 6:** Patients with ocular trauma/ocular surface lesion treated with monotherapy in the emergency setting.

Therapeutic Agents	Ocular Trauma/Ocular Surface Lesion		Ocular Trauma with Foreign Body		Penetrating Injury		Burn Due to Chemical Products	
	Mean ± SD	Median (IQR)	Mean ± SD	Median (IQR)	Mean ± SD	Median (IQR)	Mean ± SD	Median (IQR)
Topical/local treatment								
Antibiotics	55.2 ± 30.0	57.5 (30–70)	72.9 ± 30.1	80 (51.2–100)	33.0 ± 34.4	20 (0–50)	44.1 ± 34.3	13.5 (0–40)
Corticosteroids	5.3 ± 9.1	0 (0–10)	0.9 ± 3.3	0 (0–0)	1.9 ± 6.2	0 (0–0)	40.8 ± 36.3	35 (0–67.5)
Antiseptics	10.9 ± 16.6	0.5 (0–18.7)	8.1 ± 11.7	0 (0–10)	1.1 ± 3.0	0 (0–0)	11.5 ± 16.4	0 (0–23.7)
Re-epithelizing ointment	17.5 ± 20.6	10 (2.7–20)	16.9 ± 25.3		0	0	0	0
Lid wipes	1.4 ± 3.4	0 (0–0)	1.1 ± 4.2		0.5 ± 2.9		0.5 ± 2.1	
Occlusion	2.8 ± 5.5	0 (0–2)	0	0	0	0	0	0
Therapeutic contact lens	4.0 ± 8.0	0 (0–5)	0	0	0	0	0	0
Cycloplegics	0	0	0	0	2.5 ± 5.8		0	0
Systemic treatment								
Antibiotics	3.0 ± 14.7	0 (0–0)	0	0	61.1 ± 36.2	60 (32.5–100)	2.0 ± 7.8	0 (0–0)
Corticosteroids	0	0	0	0	0	0	1.0 ± 3.5	0 (0–0)

SD: standard deviation; IQR: interquartile range.

**Table 7:** Combined therapy in patients with ocular trauma/ocular surface lesion in the emergency setting.

Type of Trauma	Combined Treatment	Prescription Mean (%)
Ocular trauma with ocular surface lesion	Topical antibiotics + re-epithelizing ointment	43.4
	Topical antibiotics + re-epithelizing ointment + occlusion	40.4
Ocular trauma with foreign body	Topical antibiotics + re-epithelizing ointment	67.3
	Topical antibiotics + antiseptics + re-epithelizing ointment + lid wipes	42.5
Penetrating injury	Topical antibiotics + systemic antibiotics + topical corticosteroids + cycloplegics	65.2
	Topical antibiotics + systemic antibiotics + cycloplegics + lid wipes	55.0
Burn due to chemical products	Topical antibiotics + antiseptics	71.2
	Topical antibiotics + topical corticosteroids	66.4

The two more frequent combinations are included for each category.

## Data Availability

Study data are available from the corresponding author upon request.

## Supplementary Materials

The following supporting information can be downloaded at https://www.mdpi.com/article/10.3390/antibiotics15090856/s1, Table S1: Study questionnaire; Table S2: Checklist for Reporting Of Survey Studies (CROSS).

## Abbreviations

The following abbreviations are used in this manuscript:

AMR	Antimicrobial resistance
ANA	Antinuclear antibodies
ANCA	Anti-neutrophil cytoplasmic antibodies
CI	Confidence interval
CT	Computed tomography
FT-ABS	Fluorescent treponemal antibody absorption test
HSV	Herpes simplex virus
IOP	Intraocular pressure
IQR	Interquartile range
NA	Not applicable
PCR	Polymerase chain reaction
PHMB	Polyhexanide
PVP-I	Povidone-iodine
SBCVA	Spectacle-corrected visual acuity
SCUT	Steroids for Corneal Ulcers Trial
SD	Standard deviation
VA	Visual acuity
VZV	Varicella-zoster virus

## References

[1] Whitcher J.P., Srinivasan M., Upadhyay M.P. (2001). Corneal blindness: A global perspective. Bull. World Health Organ..

[2] Flaxman S.R., Bourne R.R.A., Resnikoff S., Ackland P., Braithwaite T., Cicinelli M.V., Das A., Jonas J.B., Keeffe J., Kempen J.H. (2017). Global causes of blindness and distance vision impairment 1990-2020: A systematic review and meta-analysis. Lancet Glob. Health.

[3] Resnikoff S., Pascolini D., Etya’ale D., Kocur I., Pararajasegaram R., Pokharel G.P., Mariotti S.P. (2004). Global data on visual impairment in the year 2002. Bull. World Health Organ..

[4] Ting D.S.J., Ho C.S., Deshmukh R., Said D.G., Dua H.S. (2021). Infectious keratitis: An update on epidemiology, causative microorganisms, risk factors, and antimicrobial resistance. Eye.

[5] Cabrera-Aguas M., Khoo P., Watson S.L. (2022). Infectious keratitis: A review. Clin. Exp. Ophthalmol..

[6] Garg D., Daigavane S. (2024). A comprehensive review on Acanthamoeba keratitis: An overview of epidemiology, risk factors, and therapeutic strategies. Cureus.

[7] Gautam M., Lal B., Patel S., Mohan R.R., Barathi A., Yadav N., Verma S.K., Nyodu R., Sampath A., Koshti D. (2025). An Emerging global threat of mycotic keratitis caused by uncommon fungal species: A systematic review and meta-analysis. Transl. Vis. Sci. Technol..

[8] Sharma S., Singla N., Arya S.K., Gulati N., Chander J. (2025). Keratomycosis: An insight into epidemiology, etiology, and antifungal susceptibility testing of causative agents at a tertiary care centre. Med. Mycol..

[9] Ting D.S.J., Cairns J., Gopal B.P., Ho C.S., Krstic L., Elsahn A., Lister M., Said D.G., Dua H.S. (2021). Risk factors, clinical outcomes, and prognostic factors of bacterial keratitis: The Nottingham Infectious Keratitis Study. Front. Med..

[10] Pelletier J., Reagan K., McLeod S., Kronk N., Dickson K., Ohman K., Santos M. (2025). Epidemiology of ocular trauma in limited-resource settings: A narrative review. Front. Med..

[11] Min H.S., Lee K.S., Sung H.K., Lee J., Kim Y.T. (2026). Epidemiology and Risk of Vision Loss in Work-Related Ocular Trauma: Evidence from a Nationwide Trauma Registry. Ophthalmic Epidemiol..

[12] Heath Jeffery R.C., Dobes J., Chen F.K. (2022). Eye injuries: Understanding ocular trauma. Aust. J. Gen. Pract..

[13] Nowrouzi-Kia B., Nadesar N., Sun Y., Gohar B., Casole J., Nowrouzi-Kia B. (2020). Types of ocular injury and their antecedent factors: A systematic review and meta-analysis. Am. J. Ind. Med..

[14] Pasricha N.D., Larco P., Miller D., Altamirano D.S., Rose-Nussbaumer J.R., Alfonso E.C., Amescua G. (2025). Infectious Keratitis Management: 10-Year Update. J. Clin. Med..

[15] Vought V., Zarbin F., Vought R., Khouri A.S. (2025). Patterns and Prevention of Occupational Eye Injuries: A Narrative Review. Clin. Ophthalmol..

[16] Woodward M.A., Vogt E.L., Niziol L.M., Mian S.I., Sugar A., Verkade A., Nallasamy N., Pawar M., Kang L., Miller K.D. (2025). Factors associated with vision outcomes in microbial keratitis: A multisite prospective cohort study. Ophthalmology.

[17] Lin A., Rhee M.K., Akpek E.K., Amescua G., Farid M., Garcia-Ferrer F.J., Varu D.M., Musch D.C., Dunn S.P., Mah F.S. (2019). American Academy of Ophthalmology Preferred Practice Pattern Cornea and External Disease Panel. Bacterial Keratitis Preferred Practice Pattern^®^. Ophthalmology.

[18] Wespiser S., Koestel E., Fabacher T., Sauer A., Bourcier T., SMIK Study Group (2023). Practice patterns in the management of bacterial keratitis: A five-continent survey. Graefes Arch. Clin. Exp. Ophthalmol..

[19] McAllum P.J., McGhee C.N. (2003). Prescribing trends in infectious keratitis: A survey of New Zealand ophthalmologists. Clin. Exp. Ophthalmol..

[20] Austin A., Schallhorn J., Geske M., Mannis M., Lietman T., Rose-Nussbaumer J. (2017). Empirical treatment of bacterial keratitis: An international survey of corneal specialists. BMJ Open Ophthalmol..

[21] Shahriari M., Mohammad M., Foruzani Haghighi M., Mohammadnezhad G., Esmaily H. (2023). Knowledge about prescribing antibiotics as prophylaxis in patients with open globe injury: A survey in Iranian ophthalmologists. Bull. Emerg. Trauma.

[22] Girbardt C., Suckert N., Hampel U. (2025). Treatment of traumatic corneal abrasion: Results of a national survey among German ophthalmologists. Ophthalmologie.

[23] Song A., Yang Y., Henein C., Bunce C., Qureshi R., Ting D.S.J. (2025). Topical antibiotics for treating bacterial keratitis: A network meta-analysis. Cochrane Database Syst. Rev..

[24] Rhee M.K., Ahmad S., Amescua G., Cheung A.Y., Choi D.S., Jhanji V., Lin A., Mian S.I., Viriya E.T., Mah F.S. (2024). Bacterial Keratitis Preferred Practice Pattern^®^. Ophthalmology.

[25] Srinivasan M., Mascarenhas J., Rajaraman R., Ravindran M., Lalitha P., Glidden D.V., Ray K.J., Hong K.C., Oldenburg C.E., Lee S.M. (2012). Corticosteroids for bacterial keratitis: The Steroids for Corneal Ulcers Trial (SCUT). Arch. Ophthalmol..

[26] Lalitha P., Srinivasan M., Rajaraman R., Ravindran M., Mascarenhas J., Priya J.L., Sy A., Oldenburg C.E., Ray K.J., Zegans M.E. (2012). Nocardia keratitis: Clinical course and effect of corticosteroids. Am. J. Ophthalmol..

[27] Srinivasan M., Mascarenhas J., Rajaraman R., Ravindran M., Lalitha P., O’Brien K.S., Glidden D.V., Ray K.J., Oldenburg C.E., Zegans M.E. (2014). The steroids for corneal ulcers trial (SCUT): Secondary 12-month clinical outcomes of a randomized controlled trial. Am. J. Ophthalmol..

[28] Ray K.J., Srinivasan M., Mascarenhas J., Rajaraman R., Ravindran M., Glidden D.V., Oldenburg C.E., Sun C.Q., Zegans M.E., McLeod S.D. (2014). Early addition of topical corticosteroids in the treatment of bacterial keratitis. JAMA Ophthalmol..

[29] White M.L., Chodosh J. Herpes Simplex Virus Keratitis: A Treatment Guideline—2014. Reviewed and Endorsed by the Ocular Microbiology and Immunology Group. https://www.aao.org/education/clinical-statement/herpes-simplex-virus-keratitis-treatment-guideline.

[30] Gu W., Zou Y., Yang M., Zhang J., Ye Z., Deng J., Zong Y., Ohno-Matsui K., Kamoi K. (2026). Varicella-zoster virus and the eye: Clinical spectrum, management, and vaccination. Pathogens.

[31] American Academy of Ophthalmology EyeWiki^®^. Varicella Zoster Virus Stromal Keratitis and Endotheliitis. https://eyewiki.org/Varicella_Zoster_Virus_Stromal_Keratitis_and_Endotheliitis#Medical_Therapy.

[32] Ng S.M., Leslie L., Tzang C.C., Algarni A.M., Kuo I.C., Lawrenson A.L., Supported by the Cochrane Eyes and Vision Review Group (2025). Antibiotic prophylaxis for corneal abrasion. Cochrane Database Syst. Rev..

[33] Bizrah M., Yusuf A., Ahmad S. (2019). An update on chemical eye burns. Eye.

[34] Osei Duah Junior I., Ampong J., Danquah C.A. (2025). Mechanisms and evolution of antimicrobial resistance in ophthalmology: Surveillance, clinical implications, and future therapies. Antibiotics.

[35] Cabrera-Aguas M., Chidi-Egboka N., Kandel H., Watson S.L. (2024). Antimicrobial resistance in ocular infection: A review. Clin. Exp. Ophthalmol..

[36] Ung L., Bispo P.J.M., Shanbhag S.S., Gilmore M.S., Chodosh J. (2019). The persistent dilemma of microbial keratitis: Global burden, diagnosis, and antimicrobial resistance. Surv. Ophthalmol..

[37] Grzybowski A., Bajoriūnaitė A., Žemaitienė R. (2025). The rising importance of antiseptics in ophthalmology: From endophthalmitis prevention to treatment of ocular infections. Ophthalmol. Ther..

[38] Martin M.J., Rahman M.R., Johnson G.J., Srinivasan M., Clayton Y.M. (1995–1996). Mycotic keratitis: Susceptibility to antiseptic agents. Int. Ophthalmol..

[39] Passidomo F., Pignatelli F., Addabbo G., Costagliola C. (2021). Topical Liposomal Ozonated Oil in Complicated Corneal Disease: A Report on Three Clinical Cases. Int. Med. Case Rep. J..

[40] Mogollón Giralt I., Boto de los Bueis A., Martín Ucero A., Vázquez Colomo P. (2025). Management of neurotrophic corneal ulcer with a topical liposomal ozonated oil: A report on three clinical cases. Am. J. Ophthalmol. Case Rep..

[41] Troisi M., Costagliola C., Rinaldi M., Strianese D., Chiariello Vecchio E., Troisi S. (2024). Efficacy and safety of Keratosept eye drops in patients with punctate keratitis: Clinical and microbiological evaluation on 50 eyes. Microorganisms.

[42] Venugopal A., Christy J., Raut V., P. P., Patwardhan V., V. V., Madkaikar A., P. M., Meenakshi R., Ramakrishnan R. (2024). Viral keratitis, surgical intervention in viral keratitis, challenges in diagnosis and treatment of viral keratitis, HSV, HZV. Semin. Ophthalmol..

[43] Awad R., Ghaith A.A., Awad K., Mamdouh Saad M., Elmassry A.A. (2024). Fungal keratitis: Diagnosis, management, and recent advances. Clin. Ophthalmol..

[44] Gurnani B., Ronquillo Y., Moshirfar M. (2023). Acanthamoeba Keratitis. StatPearls.

[45] Singh M., Husain N., Dutta S., Sinha B.P., Mohan L., Dikshit H., Sharma B. (2026). Efficacy and safety of polyhexamethylene biguanide for Acanthamoeba keratitis: Systematic review and proportional meta-analysis. Eur. J. Ophthalmol..

[46] Anbesse D.H., Yeo S., Chong B., Angell B., Stapleton F., Petsoglou C., Carnt N.A. (2025). Economic cost analysis of Acanthamoeba keratitis among contact lens wearers. Eye Contact Lens.

[47] Daley J.R., Lee M.K., Wang X., Ly M., Samarawickrama C. (2023). Epidemiology and economic cost analysis of microbial keratitis from a tertiary referral hospital in Australia. Pathogens.

[48] Sharma A., Minh Duc N.T., Luu Lam Thang T., Nam N.H., Ng S.J., Abbas K.S., Huy N.T., Marušić A., Paul C.L., Kwok J. (2021). A Consensus-Based Checklist for Reporting of Survey Studies (CROSS). J. Gen. Intern. Med..

