# Supplementary material for: Real-World Management and Patterns of Antibiotic Use in Patients with Keratitis and Ocular Surface Injuries: A Nationwide Survey Study in Spain
Source: Antibiotics (Basel). 2026 Sep 1;15(9):856. doi: 10.3390/antibiotics15090856 (PMC13603684; doi:10.3390/antibiotics15090856)
Supplement: Supplementary file 1 [file antibiotics-15-00856-s001.zip › Table S1-supplementary.pdf]

Table S1. Study questionnaire

A. Data of the investigator

1. Sex:
    - Men
    - Women

2. Age ………. years

3. Years of professional practice (since third-year fellowship) ……… years

4. How many days a week do you work in the emergency department/outpatient consultation?

B. Data of the center

5. Where do you carry out your main professional activity?
    - In a public center
    - In a private center
    - In a mixed public/private center

6. Indicate the autonomous community in which you are currently working

7. Would you please indicate the level of your hospital?
    - Level I (regional)
    - Level II (depends from other hospital for more specialized care)
    - Level III (offers all currently available diagnostic and treatment techniques)

8. Number of DIFFERENT patients that you attend in a regular month

    <u>Emergency ophthalmology</u>
    - Including all ophthalmological disorders
    - Ocular injuries and/or ocular surface lesions (new patients)
    - Ocular injuries and/or ocular surface lesions (patients on treatment)

    <u>Ocular surface ophthalmology</u>
    - Including all ophthalmological disorders
    - Keratitis (new patients)
    - Keratitis (patients on treatment)

9. Allocate 100% of your patients according to the following lesions

    <u>Emergency ophthalmology</u>
    - Patients with ocular trauma and superficial injury
    - Patients with ocular trauma and foreign body
    - Patients with keratitis/corneal ulcers due to contact lens wear
    - Patients with infectious keratitis/corneal ulcers
    - Patients with actinic keratitis (photokeratitis, sun, snow)
    - Patients with chemical burns caused by chemical agents
    - Patients with penetrating injuries
    - Patients with blunt contusions
    - Patients with non-infectious or sterile corneal ulcers
    - Patients with non-infectious keratitis

Ocular surface ophthalmology

- Patients with bacterial keratitis/corneal ulcers
- Patients with viral keratitis/corneal ulcers
- Patients with fungal keratitis/corneal ulcers
- Patients with amoebic (Acanthamoeba) keratitis/corneal ulcers
- Patients with keratitis/corneal ulcers due to contact lens wear
- Patients with non-infectious or sterile corneal ulcers
- Patients with non-infectious or sterile keratitis

C. Profile of patients with ocular surface lesion: keratitis, corneal ulcer

10. Considering all patients with ocular surface lesions: keratitis/corneal ulcers,

10.1. What is the percentage distribution by sex?"

- Men
- Women

10.2. What is the percentage distribution by age?

- < 10 years
- ≥ 10 to < 20 years
- ≥ 20 to < 40 years
- ≥ 40 to < 60 years
- ≥ 60 years

10.3. What percentage would you assign to each of these causes or predisposing factors for ocular surface lesions: keratitis/corneal ulcers

10.3.1. Ocular

- Traumatism
- Use of contact lens
- Eyelid or ocular adnexal pathology
- Ocular surface disorder: dry eye, allergy, infection, chemical lesion
- Chemical lesion, actinic
- Topical medication: corticosteroids, anesthetics, inappropriate antibiotic use …
- Ocular surgery: cataract, pterygium, keratoplasty, LASIK …
- Bullous keratopathy

10.3.2. Systemic

- Diabetes mellitus
- Sjögren's syndrome
- Stevens-Johnson syndrome
- AIDS
- Connective tissue diseases
- Malnutrition
- Oncological diseases
- Dermatological diseases (e.g., ocular rosacea)

10.3.3. Occupational

- Farmers
- Gardeners
- Animal handlers
- Metallurgical workers
- Others

10.3.4. What is the proportion of each of these causes?

- Ocular
- Systemic
- Occupational

11. “Which of these tests are routinely included in your diagnostic workup? Rate the importance of each test from 0 (not important) to 10 (very important)

Diagnostic studies (emergency ophthalmology setting)	Ocular injury	Keratitis/ corneal ulcer
Detailed external eye exam, visual acuity, pain severity evaluation		
Slit lamp: location, shape, appearance, size, margins, infiltration		
Eyelid eversion		
Fluorescein staining (+ blue light)		
Corneal sensitivity		
IOP measurement, ophthalmoscopy		
CT in penetrating, open lesions, orbital fracture		
Microbiological examination after corneal scraping: Gram, calcofluor white, and acid-fast staining …// culture media blood agar, chocolate agar, Sabouraud agar, thioglycolate broth		
Microbiological examination after corneal scraping: polymerase chain reaction (PCR)		
Visual field examination, pupillary response, optic nerve function		
Confocal microscopy		
B-mode echography		

Diagnostic studies (ocular surface consultation setting)	Keratitis	Corneal ulcer
Visual acuity, external examination of eyelids and eyelashes		
Slit lamp: location, shape, appearance, size, margins, infiltration		
Corneal sensitivity		
Fluorescein staining (+ blue light)		
Systemic studies: blood tests, urinalysis, renal function, serological tests ANA, ANCA, FT-ABS …		
CT in penetrating, open lesions, orbital fracture		
Microbiological examination after corneal scraping: Gram, calcofluor white, and acid-fast staining …		
Culture media blood agar, chocolate agar, Sabouraud agar, thioglycolate broth		
Microbiological examination after corneal scraping: polymerase chain reaction (PCR)		
Confocal microscopy		
Use of vital staining: fluor, lissamine green		

12. How easy do you find it to diagnose and differentiate between the different types of keratitis in your clinical practice? (0 = not easy at all, 10 = very easy)

Type of keratitis	Degree of ease in diagnosis or differentiation (0–10)
Bacterial keratitis	
Viral keratitis	
Fungal keratitis	
Protozoal keratitis	
Marginal keratitis	
Allergic keratoconjunctivitis	
Neutrophic keratitis	
Exposure keratopathy (lagophthalmos)	

Peripheral ulcerative keratitis	
Mooren ulcer	

13. Do you think your department in your center provides accurate diagnosis and classification of keratitis?
    - o Yes
    - o No

    13.1. If you answered NO to question 13, why?"

14. Do you think there have been any recent developments or improvements in the keratitis diagnostic process?"
    - o Yes
    - o No

    14.1. If you answered YES to question 14, which?"

D. Clinical care pathway for patients with keratitis and corneal ulcers

15. Which symptoms and signs does the patient present before entering the clinical care pathway, and what proportion of all reported symptoms do they represent?"
    - o Pain ……………………………………… %
    - o *Redness ………………………………… %*
    - o *Photophobia …………………………. %*
    - o *Foreign body sensation ……………..%*
    - o *Ocular discharge ………………………. %*
    - o *Tearing …………………………………….. %*
    - o *Eyelid swelling …………………………. %*

16. Through which healthcare professional, if any, does the patient usually enter the clinical care pathway?

    <u>Emergency ophthalmology</u>
    - o The patient themselves ……………………. %
    - o Primary care physician ………………………. %
    - o Pharmacist (community pharmacy)……… %
    - o Optician ……………………………………………. %
    - o Rheumatologist ………………………………… %
    - o Oncologist ………………………………………… %
    - o Dermatologist …………………………………… %
    - o Neurologist ………………………………………. %
    - o Another ophthalmologist ……………………. %
    - o Dissatisfied patient (second opinion) …… %
    - o Other ………………………………………………… %

    <u>Ocular surface ophthalmology</u>
    - o Ophthalmological emergencies …………… %
    - o Primary care physician ………………………. %
    - o Pharmacist (community pharmacy) …….. %
    - o Optician ……………………………………………. %
    - o Rheumatologist ………………………………… %
    - o Oncologist ………………………………………… %
    - o Dermatologist …………………………………… %
    - o Neurologist ………………………………………. %
    - o Another ophthalmologist ……………………. %

- Dissatisfied patient (second opinion) …… %

17. Please select the actions you perform (multiple choice)
    - You diagnose
    - You treat
    - You follow the patient
    - You refer

17.1. To which specialists are patients with keratitis and corneal ulcers typically referred?
17.2. In which cases?
17.3. On average, how often do you schedule follow-up appointments for the patient in each of the following cases? Please indicate the number of days.

Type pf keratitis	How many days apart do you schedule follow-ups?	How many times do you plan the follow-up?
Bacterial keratitis	days	Number of times
Viral keratitis	days	Number of times
Fungal keratitis	days	Number of times
Protozoal keratitis	days	Number of times
Infectious keratitis due to contact lens wear	days	Number of times
Actinic keratitis	days	Number of times
Vernal keratoconjunctivitis	days	Number of times
Atopic keratoconjunctivitis	days	Number of times
Neutrophic keratitis	days	Number of times
Exposure keratopathy (lagophthalmos)	days	Number of times
Peripheral ulcerative keratitis	days	Number of times
Mooren ulcer	days	Number of times

E. Treatment of keratitis

18. Please, for the remainder of this section, focus on your patients with keratitis who have received treatment (we will distinguish between each type of keratitis)

18.1. Bacterial keratitis
18.1.1. Do you use antibiotics as monotherapy or in combination in your patients with bacterial keratitis?
- Yes
- No

18.1.2. For your patients treated for bacterial keratitis, please indicate the proportion receiving each of the suggested treatments as monotherapy
- Topical antibiotics …………….%
- Antiseptics ……………………..%
- Wipes …………………………….%
- Antibiotics (systemic)……….%
- Systemic tetracyclines ……..%

18.1.3. Please indicate the combinations you use most frequently and the proportion of your patients for whom you use them

Antibiotics (topical) ☐	Antibiotics (systemic) ☐	Wipes ☐	Corticoids ☐	Antiseptics ☐	Systemic tetracyclines ☐	_____%

Antibiotics (topical) ☐	Antibiotics (systemic) ☐	Wipes ☐	Corticoids ☐	Antiseptics ☐	Systemic tetracyclines ☐	_____%
Antibiotics (topical) ☐	Antibiotics (systemic) ☐	Wipes ☐	Corticoids ☐	Antiseptics ☐	Systemic tetracyclines ☐	_____%
Antibiotics (topical) ☐	Antibiotics (systemic) ☐	Wipes ☐	Corticoids ☐	Antiseptics ☐	Systemic tetracyclines ☐	_____%
Antibiotics (topical) ☐	Antibiotics (systemic) ☐	Wipes ☐	Corticoids ☐	Antiseptics ☐	Systemic tetracyclines ☐	_____%
Antibiotics (topical) ☐	Antibiotics (systemic) ☐	Wipes ☐	Corticoids ☐	Antiseptics ☐	Systemic tetracyclines ☐	_____%
Other specify						_____%
Do not use combinations ☐						

18.1.4. Please describe below the characteristics a patient with bacterial keratitis must have for you to prescribe an antibiotic

18.1.5. Please describe below the clinical characteristics a patient with bacterial keratitis must have for you not to prescribe an antibiotic

18.2. Viral keratitis

18.2.1. Do you use antibiotics as monotherapy or in combination in your patients with viral keratitis?

- o Yes
- o No

18.2.2. For your patients treated for viral keratitis, please indicate the proportion receiving each of the suggested treatments as monotherapy

	**Treatments you use as monotherapy for herpes simplex virus (VSH) keratitis**	**Epithelial involvement % patients**	**Stromal involvement % patients**	**Endothelial involvement % patients**
☐	Antibiotics (topical)	_____%	_____%	_____%
☐	Antibiotics (systemic)	_____%	_____%	_____%
☐	Antiviral drugs (topical)	_____%	_____%	_____%
☐	Antiviral drugs (systemic)	_____%	_____%	_____%
☐	Antiseptics	_____%	_____%	_____%
☐	Corticoids (topical)	_____%	_____%	_____%
☐	Corticoids (systemic)	_____%	_____%	_____%
☐	Wipes	_____%	_____%	_____%
	Total patients	**100 %**	**100 %**	**100 %**

	**Treatments you use as monotherapy for varicella-zoster virus (VZV) keratitis**	**Epithelial involvement % patients**	**Stromal involvement % patients**	**Endothelial involvement % patients**
☐	Antibiotics (topical)	_____%	_____%	_____%
☐	Antibiotics (systemic)	_____%	_____%	_____%
☐	Antiviral drugs (topical)	_____%	_____%	_____%
☐	Antiviral drugs (systemic)	_____%	_____%	_____%
☐	Antiseptics	_____%	_____%	_____%
☐	Corticoids (topical)	_____%	_____%	_____%

☐	Corticoids (systemic)	_____%	_____%	_____%
☐	Wipes	_____%	_____%	_____%
	Total patients	**100 %**	**100 %**	**100 %**

	**Treatments do you use as monotherapy for epidemic adenoviral keratitis**	**Epithelial involvement % patients**	**Stromal involvement % patients**	**Endothelial involvement % patients**
☐	Antibiotics (topical)	_____%	_____%	_____%
☐	Antibiotics (systemic)	_____%	_____%	_____%
☐	Antiviral drugs (topical)	_____%	_____%	_____%
☐	Antiviral drugs (systemic)	_____%	_____%	_____%
☐	Antiseptics	_____%	_____%	_____%
☐	Corticoids (topical)	_____%	_____%	_____%
☐	Corticoids (systemic)	_____%	_____%	_____%
☐	Cyclosporine	_____%	_____%	_____%
☐	Wipes	_____%	_____%	_____%
	Total patients	**100 %**	**100 %**	**100 %**

18.2.3. For your patients treated for viral keratitis, please indicate the proportion receiving each of the suggested treatments as combined therapy

Herpes simplex virus (VSH) keratitis

VSH keratitis with epithelial involvement

Antibiotics (topical) ☐	Antibiotics (systemic) ☐	Antivirals (topical) ☐	Antivirals (systemic) ☐	Antiseptics ☐	Corticoids (topical) ☐	Corticoids (systemic) ☐	Wipes ☐	_____%
Antibiotics (topical) ☐	Antibiotics (systemic) ☐	Antivirals (topical) ☐	Antivirals (systemic) ☐	Antiseptics ☐	Corticoids (topical) ☐	Corticoids (systemic) ☐	Wipes ☐	_____%
Antibiotics (topical) ☐	Antibiotics (systemic) ☐	Antivirals (topical) ☐	Antivirals (systemic) ☐	Antiseptics ☐	Corticoids (topical) ☐	Corticoids (systemic) ☐	Wipes ☐	_____%
Antibiotics (topical) ☐	Antibiotics (systemic) ☐	Antivirals (topical) ☐	Antivirals (systemic) ☐	Antiseptics ☐	Corticoids (topical) ☐	Corticoids (systemic) ☐	Wipes ☐	_____%
Antibiotics (topical) ☐	Antibiotics (systemic) ☐	Antivirals (topical) ☐	Antivirals (systemic) ☐	Antiseptics ☐	Corticoids (topical) ☐	Corticoids (systemic) ☐	Wipes ☐	_____%
Antibiotics (topical) ☐	Antibiotics (systemic) ☐	Antivirals (topical) ☐	Antivirals (systemic) ☐	Antiseptics ☐	Corticoids (topical) ☐	Corticoids (systemic) ☐	Wipes ☐	_____%
Antibiotics (topical) ☐	Antibiotics (systemic) ☐	Antivirals (topical) ☐	Antivirals (systemic) ☐	Antiseptics ☐	Corticoids (topical) ☐	Corticoids (systemic) ☐	Wipes ☐	_____%
Antibiotics (topical) ☐	ANTIBIOTICS (systemic) ☐	Antivirals (topical) ☐	Antivirals (systemic) ☐	Antiseptics ☐	Corticoids (topical) ☐	Corticoids (systemic) ☐	Wipes ☐	_____%
Other specify								_____%
I do not use combinations ☐								

VSH keratitis with stromal involvement

Antibiotics (topical) ☐	Antibiotics (systemic) ☐	Antivirals (topical) ☐	Antivirals (systemic) ☐	Antiseptics ☐	Corticoids (topical) ☐	Corticoids (systemic) ☐	Wipes ☐	_____%
Antibiotics (topical) ☐	Antibiotics (systemic) ☐	Antivirals (topical) ☐	Antivirals (systemic) ☐	Antiseptics ☐	Corticoids (topical) ☐	Corticoids (systemic) ☐	Wipes ☐	_____%
Antibiotics (topical) ☐	Antibiotics (systemic) ☐	Antivirals (topical) ☐	Antivirals (systemic) ☐	Antiseptics ☐	Corticoids (topical) ☐	Corticoids (systemic) ☐	Wipes ☐	_____%

Antibiotics (topical) ☐	Antibiotics (systemic) ☐	Antivirals (topical) ☐	Antivirals (systemic) ☐	Antiseptics ☐	Corticoids (topical) ☐	Corticoids (systemic) ☐	Wipes ☐	_____%
Antibiotics (topical) ☐	Antibiotics (systemic) ☐	Antivirals (topical) ☐	Antivirals (systemic) ☐	Antiseptics ☐	Corticoids (topical) ☐	Corticoids (systemic) ☐	Wipes ☐	_____%
Antibiotics (topical) ☐	Antibiotics (systemic) ☐	Antivirals (topical) ☐	Antivirals (systemic) ☐	Antiseptics ☐	Corticoids (topical) ☐	Corticoids (systemic) ☐	Wipes ☐	_____%
Antibiotics (topical) ☐	Antibiotics (systemic) ☐	Antivirals (topical) ☐	Antivirals (systemic) ☐	Antiseptics ☐	Corticoids (topical) ☐	Corticoids (systemic) ☐	Wipes ☐	_____%
Antibiotics (topical) ☐	ANTIBIOTICS (systemic) ☐	Antivirals (topical) ☐	Antivirals (systemic) ☐	Antiseptics ☐	Corticoids (topical) ☐	Corticoids (systemic) ☐	Wipes ☐	_____%
Other specify								_____%
I do not use combinations ☐								

VSH keratitis with endothelial involvement

Antibiotics (topical) ☐	Antibiotics (systemic) ☐	Antivirals (topical) ☐	Antivirals (systemic) ☐	Antiseptics ☐	Corticoids (topical) ☐	Corticoids (systemic) ☐	Wipes ☐	_____%
Antibiotics (topical) ☐	Antibiotics (systemic) ☐	Antivirals (topical) ☐	Antivirals (systemic) ☐	Antiseptics ☐	Corticoids (topical) ☐	Corticoids (systemic) ☐	Wipes ☐	_____%
Antibiotics (topical) ☐	Antibiotics (systemic) ☐	Antivirals (topical) ☐	Antivirals (systemic) ☐	Antiseptics ☐	Corticoids (topical) ☐	Corticoids (systemic) ☐	Wipes ☐	_____%
Antibiotics (topical) ☐	Antibiotics (systemic) ☐	Antivirals (topical) ☐	Antivirals (systemic) ☐	Antiseptics ☐	Corticoids (topical) ☐	Corticoids (systemic) ☐	Wipes ☐	_____%
Antibiotics (topical) ☐	Antibiotics (systemic) ☐	Antivirals (topical) ☐	Antivirals (systemic) ☐	Antiseptics ☐	Corticoids (topical) ☐	Corticoids (systemic) ☐	Wipes ☐	_____%
Antibiotics (topical) ☐	Antibiotics (systemic) ☐	Antivirals (topical) ☐	Antivirals (systemic) ☐	Antiseptics ☐	Corticoids (topical) ☐	Corticoids (systemic) ☐	Wipes ☐	_____%
Antibiotics (topical) ☐	Antibiotics (systemic) ☐	Antivirals (topical) ☐	Antivirals (systemic) ☐	Antiseptics ☐	Corticoids (topical) ☐	Corticoids (systemic) ☐	Wipes ☐	_____%
Antibiotics (topical) ☐	ANTIBIOTICS (systemic) ☐	Antivirals (topical) ☐	Antivirals (systemic) ☐	Antiseptics ☐	Corticoids (topical) ☐	Corticoids (systemic) ☐	Wipes ☐	_____%
Other specify								_____%
I do not use combinations ☐								

Varicella-zoster virus (VZV) keratitis

Varicella-zoster virus (VZV) keratitis with epithelial involvement

Antibiotics (topical) ☐	Antibiotics (systemic) ☐	Antivirals (topical) ☐	Antivirals (systemic) ☐	Antiseptics ☐	Corticoids (topical) ☐	Corticoids (systemic) ☐	Wipes ☐	_____%
Antibiotics (topical) ☐	Antibiotics (systemic) ☐	Antivirals (topical) ☐	Antivirals (systemic) ☐	Antiseptics ☐	Corticoids (topical) ☐	Corticoids (systemic) ☐	Wipes ☐	_____%
Antibiotics (topical) ☐	Antibiotics (systemic) ☐	Antivirals (topical) ☐	Antivirals (systemic) ☐	Antiseptics ☐	Corticoids (topical) ☐	Corticoids (systemic) ☐	Wipes ☐	_____%
Antibiotics (topical) ☐	Antibiotics (systemic) ☐	Antivirals (topical) ☐	Antivirals (systemic) ☐	Antiseptics ☐	Corticoids (topical) ☐	Corticoids (systemic) ☐	Wipes ☐	_____%
Antibiotics (topical) ☐	Antibiotics (systemic) ☐	Antivirals (topical) ☐	Antivirals (systemic) ☐	Antiseptics ☐	Corticoids (topical) ☐	Corticoids (systemic) ☐	Wipes ☐	_____%

Antibiotics (topical) □	Antibiotics (systemic) □	Antivirals (topical) □	Antivirals (systemic) □	Antiseptics □	Corticoids (topical) □	Corticoids (systemic) □	Wipes □	______%
Antibiotics (topical) □	Antibiotics (systemic) □	Antivirals (topical) □	Antivirals (systemic) □	Antiseptics □	Corticoids (topical) □	Corticoids (systemic) □	Wipes □	______%
Antibiotics (topical) □	ANTIBIOTICS (systemic) □	Antivirals (topical) □	Antivirals (systemic) □	Antiseptics □	Corticoids (topical) □	Corticoids (systemic) □	Wipes □	______%
Other specify								______%
I do not use combinations □								

Varicella-zoster virus (VZV) keratitis with stromal involvement

Antibiotics (topical) □	Antibiotics (systemic) □	Antivirals (topical) □	Antivirals (systemic) □	Antiseptics □	Corticoids (topical) □	Corticoids (systemic) □	Wipes □	______%
Antibiotics (topical) □	Antibiotics (systemic) □	Antivirals (topical) □	Antivirals (systemic) □	Antiseptics □	Corticoids (topical) □	Corticoids (systemic) □	Wipes □	______%
Antibiotics (topical) □	Antibiotics (systemic) □	Antivirals (topical) □	Antivirals (systemic) □	Antiseptics □	Corticoids (topical) □	Corticoids (systemic) □	Wipes □	______%
Antibiotics (topical) □	Antibiotics (systemic) □	Antivirals (topical) □	Antivirals (systemic) □	Antiseptics □	Corticoids (topical) □	Corticoids (systemic) □	Wipes □	______%
Antibiotics (topical) □	Antibiotics (systemic) □	Antivirals (topical) □	Antivirals (systemic) □	Antiseptics □	Corticoids (topical) □	Corticoids (systemic) □	Wipes □	______%
Antibiotics (topical) □	Antibiotics (systemic) □	Antivirals (topical) □	Antivirals (systemic) □	Antiseptics □	Corticoids (topical) □	Corticoids (systemic) □	Wipes □	______%
Antibiotics (topical) □	Antibiotics (systemic) □	Antivirals (topical) □	Antivirals (systemic) □	Antiseptics □	Corticoids (topical) □	Corticoids (systemic) □	Wipes □	______%
Antibiotics (topical) □	ANTIBIOTICS (systemic) □	Antivirals (topical) □	Antivirals (systemic) □	Antiseptics □	Corticoids (topical) □	Corticoids (systemic) □	Wipes □	______%
Other specify								______%
								**100 %**
I do not use combinations □								

Varicella-zoster virus (VZV) keratitis with endothelial involvement

Antibiotics (topical) □	Antibiotics (systemic) □	Antivirals (topical) □	Antivirals (systemic) □	Antiseptics □	Corticoids (topical) □	Corticoids (systemic) □	Wipes □	______%
Antibiotics (topical) □	Antibiotics (systemic) □	Antivirals (topical) □	Antivirals (systemic) □	Antiseptics □	Corticoids (topical) □	Corticoids (systemic) □	Wipes □	______%
Antibiotics (topical) □	Antibiotics (systemic) □	Antivirals (topical) □	Antivirals (systemic) □	Antiseptics □	Corticoids (topical) □	Corticoids (systemic) □	Wipes □	______%
Antibiotics (topical) □	Antibiotics (systemic) □	Antivirals (topical) □	Antivirals (systemic) □	Antiseptics □	Corticoids (topical) □	Corticoids (systemic) □	Wipes □	______%
Antibiotics (topical) □	Antibiotics (systemic) □	Antivirals (topical) □	Antivirals (systemic) □	Antiseptics □	Corticoids (topical) □	Corticoids (systemic) □	Wipes □	______%
Antibiotics (topical) □	Antibiotics (systemic) □	Antivirals (topical) □	Antivirals (systemic) □	Antiseptics □	Corticoids (topical) □	Corticoids (systemic) □	Wipes □	______%
Antibiotics (topical) □	Antibiotics (systemic) □	Antivirals (topical) □	Antivirals (systemic) □	Antiseptics □	Corticoids (topical) □	Corticoids (systemic) □	Wipes □	______%
Antibiotics (topical) □	ANTIBIOTICS (systemic) □	Antivirals (topical) □	Antivirals (systemic) □	Antiseptics □	Corticoids (topical) □	Corticoids (systemic) □	Wipes □	______%

Other specify				______%
I do not use combinations ☐				

Adenoviral keratitis

Adenoviral keratitis with epithelial involvement

Antibiotics (topical) □	Antibiotics (systemic) □	Antivirals (topical) □	Antivirals (systemic) □	Antiseptics □	Corticoids (topical) □	Corticoids (systemic) □	Wipes □	______%
Antibiotics (topical) □	Antibiotics (systemic) □	Antivirals (topical) □	Antivirals (systemic) □	Antiseptics □	Corticoids (topical) □	Corticoids (systemic) □	Wipes □	______%
Antibiotics (topical) □	Antibiotics (systemic) □	Antivirals (topical) □	Antivirals (systemic) □	Antiseptics □	Corticoids (topical) □	Corticoids (systemic) □	Wipes □	______%
Antibiotics (topical) □	Antibiotics (systemic) □	Antivirals (topical) □	Antivirals (systemic) □	Antiseptics □	Corticoids (topical) □	Corticoids (systemic) □	Wipes □	______%
Antibiotics (topical) □	Antibiotics (systemic) □	Antivirals (topical) □	Antivirals (systemic) □	Antiseptics □	Corticoids (topical) □	Corticoids (systemic) □	Wipes □	______%
Antibiotics (topical) □	Antibiotics (systemic) □	Antivirals (topical) □	Antivirals (systemic) □	Antiseptics □	Corticoids (topical) □	Corticoids (systemic) □	Wipes □	______%
Antibiotics (topical) □	Antibiotics (systemic) □	Antivirals (topical) □	Antivirals (systemic) □	Antiseptics □	Corticoids (topical) □	Corticoids (systemic) □	Wipes □	______%
Antibiotics (topical) □	ANTIBIOTICS (systemic) □	Antivirals (topical) □	Antivirals (systemic) □	Antiseptics □	Corticoids (topical) □	Corticoids (systemic) □	Wipes □	______%
Other specify								______%
I do not use combinations ☐								

Adenoviral keratitis with stromal involvement

Antibiotics (topical) □	Antibiotics (systemic) □	Antivirals (topical) □	Antivirals (systemic) □	Antiseptics □	Corticoids (topical) □	Corticoids (systemic) □	Wipes □	______%
Antibiotics (topical) □	Antibiotics (systemic) □	Antivirals (topical) □	Antivirals (systemic) □	Antiseptics □	Corticoids (topical) □	Corticoids (systemic) □	Wipes □	______%
Antibiotics (topical) □	Antibiotics (systemic) □	Antivirals (topical) □	Antivirals (systemic) □	Antiseptics □	Corticoids (topical) □	Corticoids (systemic) □	Wipes □	______%
Antibiotics (topical) □	Antibiotics (systemic) □	Antivirals (topical) □	Antivirals (systemic) □	Antiseptics □	Corticoids (topical) □	Corticoids (systemic) □	Wipes □	______%
Antibiotics (topical) □	Antibiotics (systemic) □	Antivirals (topical) □	Antivirals (systemic) □	Antiseptics □	Corticoids (topical) □	Corticoids (systemic) □	Wipes □	______%
Antibiotics (topical) □	Antibiotics (systemic) □	Antivirals (topical) □	Antivirals (systemic) □	Antiseptics □	Corticoids (topical) □	Corticoids (systemic) □	Wipes □	______%
Antibiotics (topical) □	Antibiotics (systemic) □	Antivirals (topical) □	Antivirals (systemic) □	Antiseptics □	Corticoids (topical) □	Corticoids (systemic) □	Wipes □	______%
Antibiotics (topical) □	ANTIBIOTICS (systemic) □	Antivirals (topical) □	Antivirals (systemic) □	Antiseptics □	Corticoids (topical) □	Corticoids (systemic) □	Wipes □	______%
Other specify								______%
I do not use combinations ☐								

Adenoviral keratitis with endothelial involvement

Antibiotics (topical) ☐	Antibiotics (systemic) ☐	Antivirals (topical) ☐	Antivirals (systemic) ☐	Antiseptics ☐	Corticoids (topical) ☐	Corticoids (systemic) ☐	Wipes ☐	_____%
Antibiotics (topical) ☐	Antibiotics (systemic) ☐	Antivirals (topical) ☐	Antivirals (systemic) ☐	Antiseptics ☐	Corticoids (topical) ☐	Corticoids (systemic) ☐	Wipes ☐	_____%
Antibiotics (topical) ☐	Antibiotics (systemic) ☐	Antivirals (topical) ☐	Antivirals (systemic) ☐	Antiseptics ☐	Corticoids (topical) ☐	Corticoids (systemic) ☐	Wipes ☐	_____%
Antibiotics (topical) ☐	Antibiotics (systemic) ☐	Antivirals (topical) ☐	Antivirals (systemic) ☐	Antiseptics ☐	Corticoids (topical) ☐	Corticoids (systemic) ☐	Wipes ☐	_____%
Antibiotics (topical) ☐	Antibiotics (systemic) ☐	Antivirals (topical) ☐	Antivirals (systemic) ☐	Antiseptics ☐	Corticoids (topical) ☐	Corticoids (systemic) ☐	Wipes ☐	_____%
Antibiotics (topical) ☐	Antibiotics (systemic) ☐	Antivirals (topical) ☐	Antivirals (systemic) ☐	Antiseptics ☐	Corticoids (topical) ☐	Corticoids (systemic) ☐	Wipes ☐	_____%
Antibiotics (topical) ☐	Antibiotics (systemic) ☐	Antivirals (topical) ☐	Antivirals (systemic) ☐	Antiseptics ☐	Corticoids (topical) ☐	Corticoids (systemic) ☐	Wipes ☐	_____%
Antibiotics (topical) ☐	ANTIBIOTICS (systemic) ☐	Antivirals (topical) ☐	Antivirals (systemic) ☐	Antiseptics ☐	Corticoids (topical) ☐	Corticoids (systemic) ☐	Wipes ☐	_____%
Other specify								_____%
I do not use combinations ☐								

18.2.4. Please, describe the characteristics that a patient with viral keratitis should met for prescribing antibiotics

18.2.5. Please, describe the characteristics that a patient with viral keratitis should met for NOT prescribing antibiotics

18.3. Fungal keratitis

18.3.1. Do you use antibiotics as monotherapy or in combination in your patients with fungal keratitis?

- o Yes
- o No

18.3.2. For your patients treated for fungal keratitis, please indicate the proportion receiving each of the suggested treatments as monotherapy

	Treatments do you use as monotherapy	% Patients
☐	Antibiotics (topical)	_____%
☐	Antibiotics (systemic)	_____%
☐	Corticoids (topical)	_____%
☐	Corticoids (systemic)	_____%
☐	Wipes	_____%
☐	Antiseptics	_____%
☐	Corticoids (systemic)	_____%
☐	Antifungals: topical (natamycin, amphotericin, azoles)	_____%
☐	Antifungals: systemic: (natamycin, amphotericin, azoles)	_____%
☐	Total patients	**100**%

18.3.3. For your patients treated for fungal keratitis, please indicate the proportion receiving each of the suggested treatments as combined therapy

Antibiotics (topical) ☐	Antibiotics (systemic) ☐	Antiseptics ☐	Wipes ☐	Corticoids (systemic) ☐	Corticoids (topical) ☐	Antifungals (natamycin, amphotericin, azoles) (topical) ☐	Antifungals (natamycin, amphotericin, azoles) (systemic) ☐	_____%

Antibiotics (topical) □	Antibiotics (systemic) □	Antiseptics □	Wipes □	Corticoids (systemic) □	Corticoids (topical) □	Antifungals (natamycin, amphotericin, azoles) (topical) □	Antifungals (natamycin, amphotericin, azoles) (systemic) □	_____%
Antibiotics (topical) □	Antibiotics (systemic) □	Antiseptics □	Wipes □	Corticoids (systemic) □	Corticoids (topical) □	Antifungals (natamycin, amphotericin, azoles) (topical) □	Antifungals (natamycin, amphotericin, azoles) (systemic) □	_____%
Antibiotics (topical) □	Antibiotics (systemic) □	Antiseptics □	Wipes □	Corticoids (systemic) □	Corticoids (topical) □	Antifungals (natamycin, amphotericin, azoles) (topical) □	Antifungals (natamycin, amphotericin, azoles) (systemic) □	_____%
Antibiotics (topical) □	Antibiotics (systemic) □	Antiseptics □	Wipes □	Corticoids (systemic) □	Corticoids (topical) □	Antifungals (natamycin, amphotericin, azoles) (topical) □	Antifungals (natamycin, amphotericin, azoles) (systemic) □	_____%
Antibiotics (topical) □	Antibiotics (systemic) □	Antiseptics □	Wipes □	Corticoids (systemic) □	Corticoids (topical) □	Antifungals (natamycin, amphotericin, azoles) (topical) □	Antifungals (natamycin, amphotericin, azoles) (systemic) □	_____%
Antibiotics (topical) □	Antibiotics (systemic) □	Antiseptics □	Wipes □	Corticoids (systemic) □	Corticoids (topical) □	Antifungals (natamycin, amphotericin, azoles) (topical) □	Antifungals (natamycin, amphotericin, azoles) (systemic) □	_____%
Antibiotics (topical) □	Antibiotics (systemic) □	Antiseptics □	Wipes □	Corticoids (systemic) □	Corticoids (topical) □	Antifungals (natamycin, amphotericin, azoles) (topical) □	Antifungals (natamycin, amphotericin, azoles) (systemic) □	_____%
Other specify								_____%
I do not use combinations □								

18.3.4. Please, describe the characteristics that a patient with fungal keratitis should met for prescribing antifungals

18.3.5. Please, describe the characteristics that a patient with viral keratitis should met for NOT prescribing antifungals

18.4. Bacterial keratitis due to contact lens wear

18.4.1. Do you use antibiotics as monotherapy or in combination in your patients with keratitis due to contact lens wear?

- o Yes
- o No

18.4.2. For your patients treated for bacterial keratitis due to contact lens wear, please indicate the proportion receiving each of the suggested treatments as monotherapy

	Treatments do you use as monotherapy	% pacientes
☐	Antibiotics (topical)	_____%
☐	Antibiotics (systemic)	_____%
☐	Wipes	_____%
☐	Antiseptics	_____%
☐	Re-epithelizing ointment	_____%
☐	Artificial tears	_____%
	Total patients treated on monotherapy	**100 %**

18.4.3. For your patients treated for keratitis due to contact lens wear, please indicate the proportion receiving each of the suggested treatments as combined therapy

Antibiotics (topical) □	Antibiotics (systemic) □	Wipes □	Antiseptics □	Re-epithelizing ointment □	Artificial tears □	_____%
Antibiotics (topical) □	Antibiotics (systemic) □	Wipes □	Antiseptics □	Re-epithelizing ointment □	Artificial tears □	_____%
Antibiotics (topical) □	Antibiotics (systemic) □	Wipes □	Antiseptics □	Re-epithelizing ointment □	Artificial tears □	_____%
Antibiotics (topical) □	Antibiotics (systemic) □	Wipes □	Antiseptics □	Re-epithelizing ointment □	Artificial tears □	_____%
Antibiotics (topical) □	Antibiotics (systemic) □	Wipes □	Antiseptics □	Re-epithelizing ointment □	Artificial tears □	_____%
Antibiotics (topical) □	Antibiotics (systemic) □	Wipes □	Antiseptics □	Re-epithelizing ointment □	Artificial tears □	_____%
Other specify						_____%
I do not use combination □						

18.4.4. Please, describe the characteristics that a patient with keratitis due to contact lens wear should met for prescribing antifungals

18.4.5. Please, describe the characteristics that a patient with keratitis due to contact lens wear should met for NOT prescribing antifungals

18.5. Acanthamoeba keratitis due to contact lens wear

18.5.1. Do you use antibiotics as monotherapy or in combination in your patients with Acanthamoeba keratitis due to contact lens wear?

- o Yes
- o No

18.5.2. For your patients treated for Acanthamoeba keratitis due to contact lens wear, please indicate the proportion receiving each of the suggested treatments as monotherapy

	Treatments do you use as monotherapy	% pacientes
☐	Chlorhexidine eye drops	_____%
☐	Polyhexanide (PHMB) eye drops	_____%
☐	Propamidine eye drops (Brolene®)	_____%
☐	Hexamidine eye drops	_____%
☐	Corticoids eye drops	_____%

☐	Antibiotic eye drops	_____%
☐	Oral antifungals	_____%
	Total patients treated on monotherapy	**100 %**

18.5.3. For your patients treated for Acanthamoeba keratitis due to contact lens wear, please indicate the proportion receiving each of the suggested treatments as combined therapy

Chlorhexidine eye drops □	PHMB eye drops □	Propamidine eye drops □	Hexamidine eye drops □	Corticoids eye drops □	Antibiotic eye drops □	Oral antifungals □	_____%
Chlorhexidine eye drops □	PHMB eye drops □	Propamidine eye drops □	Hexamidine eye drops □	Corticoids eye drops □	Antibiotic eye drops □	Oral antifungals □	_____%
Chlorhexidine eye drops □	PHMB eye drops □	Propamidine eye drops □	Hexamidine eye drops □	Corticoids eye drops □	Antibiotic eye drops □	Oral antifungals □	_____%
Chlorhexidine eye drops □	PHMB eye drops □	Propamidine eye drops □	Hexamidine eye drops □	Corticoids eye drops □	Antibiotic eye drops □	Oral antifungals □	_____%
Chlorhexidine eye drops □	PHMB eye drops □	Propamidine eye drops □	Hexamidine eye drops □	Corticoids eye drops □	Antibiotic eye drops □	Oral antifungals □	_____%
Chlorhexidine eye drops □	PHMB eye drops □	Propamidine eye drops □	Hexamidine eye drops □	Corticoids eye drops □	Antibiotic eye drops □	Oral antifungals □	_____%
Chlorhexidine eye drops □	PHMB eye drops □	Propamidine eye drops □	Hexamidine eye drops □	Corticoids eye drops □	Antibiotic eye drops □	Oral antifungals □	_____%
Other specify							_____%
I do not use combinations □							

18.5.4. Please, describe the characteristics that a patient with Acanthamoeba keratitis due to contact lens wear should met for prescribing an antibiotic

18.5.5. Please, describe the characteristics that a patient with Acanthamoeba keratitis due to contact lens wear should met for NOT prescribing an antibiotic

18.6. Actinic keratitis (photokeratitis, sun, snow)

18.6.1. Do you use antibiotics as monotherapy or in combination in your patients with actinic keratitis?

- o Yes
- o No

18.6.2. For your patients treated for actinic keratitis, please indicate the proportion receiving each of the suggested treatments as monotherapy

	**Treatments do you use as monotherapy**	**% Patients**
☐	Antibiotics (topical)	_____%
☐	Antibiotics (systemic)	_____%
☐	Corticoids (topical)	_____%
☐	Antiseptics	_____%
☐	Re-epithelizing ointment	_____%
☐	Artificial tears	_____%
☐	Cycloplegics	_____%
☐	Other systemic analgesics	_____%
	Total patients treated on monotherapy	**100**%

18.6.3. For your patients treated for actinic keratitis, please indicate the proportion receiving each of the suggested treatments as combined therapy

Antibiotics (topical) □	Antibiotics (systemic) □	Corticoids (topical) □	Antiseptics □	Re-epithelizing ointment □	Artificial tears □	Cycloplegic □	Other systemic analgesics □	_____%
Antibiotics (topical) □	Antibiotics (systemic) □	Corticoids (topical) □	Antiseptics □	Re-epithelizing ointment □	Artificial tears □	Cycloplegic □	Other systemic analgesics □	_____%
Antibiotics (topical) □	Antibiotics (systemic) □	Corticoids (topical) □	Antiseptics □	Re-epithelizing ointment □	Artificial tears □	Cycloplegic □	Other systemic analgesics □	_____%
Antibiotics (topical) □	Antibiotics (systemic) □	Corticoids (topical) □	Antiseptics □	Re-epithelizing ointment □	Artificial tears □	Cycloplegic □	Other systemic analgesics □	_____%
Antibiotics (topical) □	Antibiotics (systemic) □	Corticoids (topical) □	Antiseptics □	Re-epithelizing ointment □	Artificial tears □	Cycloplegic □	Other systemic analgesics □	_____%
Antibiotics (topical) □	Antibiotics (systemic) □	Corticoids (topical) □	Antiseptics □	Re-epithelizing ointment □	Artificial tears □	Cycloplegic □	Other systemic analgesics □	_____%
Antibiotics (topical) □	Antibiotics (systemic) □	Corticoids (topical) □	Antiseptics □	Re-epithelizing ointment □	Artificial tears □	Cycloplegic □	Other systemic analgesics □	_____%
Antibiotics (topical) □	Antibiotics (systemic) □	Corticoids (topical) □	Antiseptics □	Re-epithelizing ointment □	Artificial tears □	Cycloplegic □	Other systemic analgesics □	_____%
Other specify:								_____%
I do not use combinations □								

18.6.4. Please, describe the characteristics that a patient with actinic keratitis due to contact lens wear should met for prescribing an antibiotic

18.6.5. Please, describe the characteristics that a patient with actinic keratitis should met for NOT prescribing an antibiotic

F. Drivers in the treatment of keratitis

19. The following list includes some characteristics that are attributed to treatments for keratitis. Please assess the importance that each of them has for you when initiating a treatment. Use a 0-10 scale from 0 (not important) to 10 (very important)

Item		Importance
1	Convenient dosing regimen for patients	
2	Antibacterial, antiviral, and antifungal efficacy	
3	That offers an alternative to the use of antibiotics	
4	Useful in reducing and achieving closure of the ulcer without causing haze	
5	Contributes to reduce microorganisms on the ocular surface	
6	With adjuvant activity that enhances the action of the antibiotic by reducing microbial resistance to the antibiotic	
7	That offers a greater volume of high-quality scientific evidence	
8	Hydrating and relieving action of the product	
9	Favorable evidence in clinical practice/real-world data	
10	Recommended by an expert whom I consider more respected or influential	

11	Cost-effective	
12	Stimulates corneal regeneration and has anti-inflammatory activity	
13	Indirect antioxidant activity	
14	Achieve the same result as antibiotics, but using an active ingredient that is not an antibiotic	

20. Which would you say are the MAIN BARRIERS when initiating treatments for keratitis?

21. Specifically, thinking about the barriers when prescribing treatments for keratitis, how important is the cost for you? Please use a scale from 0 to 10, where 0 = not important at all and 10 = very important.

0	1	2	3	4	5	6	7	8	9	10

22. How important do you think the cost of keratitis treatments is as a barrier for the following types of patients? Please use a scale from 0 to 10, where 0 = not important at all and 10 = very important.

	IMPORTANCIA
Patients with bacterial, viral or fungal keratitis	
Patients with infectious corneal ulcers	
Patients with non-infectious intercurrent corneal ulcers with dermatological, rheumatological or neurological diseases	

G. Clinical care pathway of patients with ocular trauma/ocular surface lesion from the emergency department

Emergency ophthalmology setting

23. Patient access to the pathway. What symptoms and signs does the patient experience before accessing the pathway, and what percentage do they represent of the total symptoms?

☐	1. Pain	_____%
☐	2. Redness	_____%
☐	3. Photophobia	_____%
☐	4. Foreign body sensation	_____%
☐	5. Ocular discharge	_____%
☐	6. Tearing	_____%
☐	7. Decrease of visual acuity	_____%
☐	8. Eyelid swelling	_____%
☐	9. Headache, nauseas	_____%

24. Through which healthcare professional, if any, does the patient usually access the pathway, and what percentage do they represent among those seen in the Emergency

☐	1. The patient themselves	_____%
☐	2. Primary care physician	_____%
☐	3. Pharmacist (community pharmacy)	_____%
☐	4. Optician	_____%
☐	5. Another ophthalmologist	_____%
☐	6. Other: _________________	_____%
	TOTAL	100 %

25. Select the actions you perform in your patients with ocular trauma and ocular surface lesions

1. You diagnose	☐

2. You treat	☐
3. You follow the patient	☐
4. You refer	☐

25.1. To which specialist would you refer your patients with ocular trauma and ocular surface lesions?

25.2. In which cases?

25.3. On average, how often do you schedule follow-up appointments for the patient in each of the following cases? Please indicate the number of days.

Type pf keratitis	How many days apart do you schedule follow-ups?	How many times do you plan the follow-up?
1. Trauma with lesion of the ocular surface	days	Number of times
2. Trauma with foreign body	days	Number of times
3. Trauma with penetrating injury of the eye globe	days	Number of times
4. Burns due to chemical products	days	Number of times
5. Blunt contusion	days	Number of times

H. Treatment of ocular trauma/ocular surface lesion

Emergency ophthalmology setting

26. Ocular trauma with lesion of the ocular surface

26.1.1. Do you use antibiotics as monotherapy or in combination in your patients with ocular trauma with lesion of the ocular surface?

- o Yes
- o No

26.1.2. For your patients treated for ocular trauma with lesion of the ocular surface, please indicate the proportion receiving each of the suggested treatments as monotherapy

	**Treatments do you use as monotherapy**	**% Patients**
☐	Antibiotics (topical)	_____%
☐	Antibiotics (systemic)	_____%
☐	Corticoids (topical)	_____%
☐	Antiseptics	_____%
☐	Re-epithelizing ointment	_____%
☐	Wipes	_____%
☐	Occlusion	_____%
☐	Therapeutic contact lens	_____%
	Total patients treated on monotherapy	**100**%

26.1.3. For your patients treated for ocular trauma with lesion of the ocular surface, please indicate the proportion receiving each of the suggested treatments as combined therapy

Antibiotics (topical) ☐	Antibiotics (systemic) ☐	Corticoids (topical) ☐	Antiseptics ☐	Re-epithelizing ointment ☐	Wipes ☐	Occlusion ☐	Therapeutic contact lens ☐	_____%
Antibiotics (topical) ☐	Antibiotics (systemic) ☐	Corticoids (topical) ☐	Antiseptics ☐	Re-epithelizing ointment ☐	Wipes ☐	Occlusion ☐	Therapeutic contact lens ☐	_____%
Antibiotics (topical) ☐	Antibiotics (systemic) ☐	Corticoids (topical) ☐	Antiseptics ☐	Re-epithelizing ointment ☐	Wipes ☐	Occlusion ☐	Therapeutic contact lens ☐	_____%
Antibiotics (topical)	Antibiotics (systemic)	Corticoids (topical)	Antiseptics ☐	Re-epithelizing ointment	Wipes ☐	Occlusion ☐	Therapeutic contact lens	_____%

□	□	□		□			□	
Antibiotics (topical) □	Antibiotics (systemic) □	Corticoids (topical) □	Antiseptics □	Re-epithelizing ointment □	Wipes □	Occlusion □	Therapeutic contact lens □	_____%
Antibiotics (topical) □	Antibiotics (systemic) □	Corticoids (topical) □	Antiseptics □	Re-epithelizing ointment □	Wipes □	Occlusion □	Therapeutic contact lens □	_____%
Antibiotics (topical) □	Antibiotics (systemic) □	Corticoids (topical) □	Antiseptics □	Re-epithelizing ointment □	Wipes □	Occlusion □	Therapeutic contact lens □	_____%
Antibiotics (topical) □	Antibiotics (systemic) □	Corticoids (topical) □	Antiseptics □	Re-epithelizing ointment □	Wipes □	Occlusion □	Therapeutic contact lens □	_____%
Other specify:								_____%
I do not use combinations □								

26.1.3.1. Please, describe the characteristics that a patient with ocular trauma with lesion of the ocular surface should met for prescribing an antibiotic

26.1.3.2. Please, describe the characteristics that a patient with ocular trauma with lesion of the ocular surface should met for NOT prescribing an antibiotic

26.2. <u>Ocular trauma with foreign body</u>

26.2.1. Do you use antibiotics as monotherapy or in combination in your patients with ocular trauma with foreign body?

- o Yes
- o No

26.2.2. For your patients treated for ocular trauma with foreign body, please indicate the proportion receiving each of the suggested treatments as monotherapy

	**Treatments do you use as monotherapy**	**% Patients**
☐	Antibiotics (topical)	_____%
☐	Antibiotics (systemic)	_____%
☐	Corticoids (topical)	_____%
☐	Antiseptics	_____%
☐	Re-epithelizing ointment	_____%
☐	Wipes	_____%
☐	Occlusion	_____%
☐	Therapeutic contact lens	_____%
	Total patients treated on monotherapy	**100**%

26.1.3. For your patients treated for ocular trauma with foreign body, please indicate the proportion receiving each of the suggested treatments as combined therapy

Antibiotics (topical) □	Antibiotics (systemic) □	Corticoids (topical) □	Antiseptics □	Re-epithelizing ointment □	Wipes □	Occlusion □	Therapeutic contact lens □	_____%
Antibiotics (topical) □	Antibiotics (systemic) □	Corticoids (topical) □	Antiseptics □	Re-epithelizing ointment □	Wipes □	Occlusion □	Therapeutic contact lens □	_____%
Antibiotics (topical) □	Antibiotics (systemic) □	Corticoids (topical) □	Antiseptics □	Re-epithelizing ointment □	Wipes □	Occlusion □	Therapeutic contact lens □	_____%
Antibiotics (topical) □	Antibiotics (systemic) □	Corticoids (topical) □	Antiseptics □	Re-epithelizing ointment □	Wipes □	Occlusion □	Therapeutic contact lens □	_____%
Antibiotics (topical) □	Antibiotics (systemic) □	Corticoids (topical) □	Antiseptics □	Re-epithelizing ointment □	Wipes □	Occlusion □	Therapeutic contact lens □	_____%

Antibiotics (topical) □	Antibiotics (systemic) □	Corticoids (topical) □	Antiseptics □	Re-epithelizing ointment □	Wipes □	Occlusion □	Therapeutic contact lens □	_____%
Antibiotics (topical) □	Antibiotics (systemic) □	Corticoids (topical) □	Antiseptics □	Re-epithelizing ointment □	Wipes □	Occlusion □	Therapeutic contact lens □	_____%
Antibiotics (topical) □	Antibiotics (systemic) □	Corticoids (topical) □	Antiseptics □	Re-epithelizing ointment □	Wipes □	Occlusion □	Therapeutic contact lens □	_____%
Other specify:								_____%
I do not use combinations □								

26.1.3.1. Please, describe the characteristics that a patient with ocular trauma with foreign body should met for prescribing an antibiotic

26.1.3.2. Please, describe the characteristics that a patient with ocular trauma with foreign body should met for NOT prescribing an antibiotic

26.3. <u>Ocular trauma with penetrating injury of the eye globe</u>

26.3.1. Do you use antibiotics as monotherapy or in combination in your patients with ocular trauma with penetrating injury of the eye globe?

- o Yes
- o No

26.3.2. For your patients treated for ocular trauma with penetrating injury of the eye globe, please indicate the proportion receiving each of the suggested treatments as monotherapy

	**Treatments do you use as monotherapy**	**% Patients**
☐	Antibiotics (topical)	_____%
☐	Antibiotics (systemic)	_____%
☐	Corticoids (topical)	_____%
☐	Antiseptics	_____%
☐	Re-epithelizing ointment	_____%
☐	Wipes	_____%
☐	Occlusion	_____%
☐	Therapeutic contact lens	_____%
	Total patients treated on monotherapy	**100**%

26.3.3. For your patients treated for ocular trauma with penetrating injury of the eye globe, please indicate the proportion receiving each of the suggested treatments as combined therapy

Antibiotics (topical) □	Antibiotics (systemic) □	Corticoids (topical) □	Antiseptics □	Re-epithelizing ointment □	Wipes □	Occlusion □	Therapeutic contact lens □	_____%
Antibiotics (topical) □	Antibiotics (systemic) □	Corticoids (topical) □	Antiseptics □	Re-epithelizing ointment □	Wipes □	Occlusion □	Therapeutic contact lens □	_____%
Antibiotics (topical) □	Antibiotics (systemic) □	Corticoids (topical) □	Antiseptics □	Re-epithelizing ointment □	Wipes □	Occlusion □	Therapeutic contact lens □	_____%
Antibiotics (topical) □	Antibiotics (systemic) □	Corticoids (topical) □	Antiseptics □	Re-epithelizing ointment □	Wipes □	Occlusion □	Therapeutic contact lens □	_____%
Antibiotics (topical) □	Antibiotics (systemic) □	Corticoids (topical) □	Antiseptics □	Re-epithelizing ointment □	Wipes □	Occlusion □	Therapeutic contact lens □	_____%

Antibiotics (topical) □	Antibiotics (systemic) □	Corticoids (topical) □	Antiseptics □	Re-epithelizing ointment □	Wipes □	Occlusion □	Therapeutic contact lens □	_____%
Antibiotics (topical) □	Antibiotics (systemic) □	Corticoids (topical) □	Antiseptics □	Re-epithelizing ointment □	Wipes □	Occlusion □	Therapeutic contact lens □	_____%
Antibiotics (topical) □	Antibiotics (systemic) □	Corticoids (topical) □	Antiseptics □	Re-epithelizing ointment □	Wipes □	Occlusion □	Therapeutic contact lens □	_____%
Other specify:								_____%
I do not use combinations □								

26.3.3.1. Please, describe the characteristics that a patient with ocular trauma with penetrating injury of the eye globe should met for prescribing an antibiotic

26.3.3.2. Please, describe the characteristics that a patient with ocular trauma with penetrating injury of the eye globe should met for NOT prescribing an antibiotic

26.4. Burns due to chemical products

26.4.1. Do you use antibiotics as monotherapy or in combination in your patients with Burns due to chemical products?

- o Yes
- o No

26.4.2. For your patients treated for burns due to chemical products, please indicate the proportion receiving each of the suggested treatments as monotherapy

	**Treatments do you use as monotherapy**	**% Patients**
☐	Antibiotics (topical)	_____%
☐	Antibiotics (systemic)	_____%
☐	Corticoids (topical)	_____%
☐	Antiseptics	_____%
☐	Re-epithelizing ointment	_____%
☐	Wipes	_____%
☐	Occlusion	_____%
☐	Therapeutic contact lens	_____%
	Total patients treated on monotherapy	**100**%

26.4.3. For your patients treated for burns due to chemical products, please indicate the proportion receiving each of the suggested treatments as combined therapy

Antibiotics (topical) □	Antibiotics (systemic) □	Corticoids (topical) □	Antiseptics □	Re-epithelizing ointment □	Wipes □	Occlusion □	Therapeutic contact lens □	_____%
Antibiotics (topical) □	Antibiotics (systemic) □	Corticoids (topical) □	Antiseptics □	Re-epithelizing ointment □	Wipes □	Occlusion □	Therapeutic contact lens □	_____%
Antibiotics (topical) □	Antibiotics (systemic) □	Corticoids (topical) □	Antiseptics □	Re-epithelizing ointment □	Wipes □	Occlusion □	Therapeutic contact lens □	_____%
Antibiotics (topical) □	Antibiotics (systemic) □	Corticoids (topical) □	Antiseptics □	Re-epithelizing ointment □	Wipes □	Occlusion □	Therapeutic contact lens □	_____%
Antibiotics (topical) □	Antibiotics (systemic) □	Corticoids (topical) □	Antiseptics □	Re-epithelizing ointment □	Wipes □	Occlusion □	Therapeutic contact lens □	_____%
Antibiotics (topical) □	Antibiotics (systemic) □	Corticoids (topical) □	Antiseptics □	Re-epithelizing ointment □	Wipes □	Occlusion □	Therapeutic contact lens □	_____%

Antibiotics (topical) □	Antibiotics (systemic) □	Corticoids (topical) □	Antiseptics □	Re-epithelizing ointment □	Wipes □	Occlusion □	Therapeutic contact lens □	_____%
Antibiotics (topical) □	Antibiotics (systemic) □	Corticoids (topical) □	Antiseptics □	Re-epithelizing ointment □	Wipes □	Occlusion □	Therapeutic contact lens □	_____%
Other specify:								_____%
I do not use combinations □								

26.4.3.1. Please, describe the characteristics that a patient with burns due to chemical products should met for prescribing an antibiotic

26.4.3.2. Please, describe the characteristics that a patient with burns due to chemical products should met for NOT prescribing an antibiotic

I. Drivers in the management of ocular trauma/ocular surface lesion

27. The following list includes some characteristics that are attributed to treatments for ocular trauma and lesion of the ocular surface. Please assess the importance that each of them has for you when initiating a treatment. Use a 0-10 scale from 0 (not important) to 10 (very important)

Item		Importance
1	Convenient dosing regimen for patients	
2	Antibacterial, antiviral, and antifungal efficacy	
3	That offers an alternative to the use of antibiotics	
4	Useful in reducing and achieving closure of the ulcer without causing haze	
5	Contributes to reduce microorganisms on the ocular surface	
6	With adjuvant activity that enhances the action of the antibiotic by reducing microbial resistance to the antibiotic	
7	That offers a greater volume of high-quality scientific evidence	
8	Hydrating and relieving action of the product	
9	Favorable evidence in clinical practice/real-world data	
10	Recommended by an expert whom I consider more respected or influential	
11	Cost-effective	
12	Gel presentation with high permanence and regeneration of the damages tissue	
13	Stimulates corneal regeneration and has anti-inflammatory activity	
14	Indirect antioxidant activity	

Ocular surface ophthalmology

27.1. Which would you say are the MAIN BARRIERS when initiating treatments for keratitis?

27.1. Specifically, thinking about the barriers when prescribing treatments for keratitis, how important is the cost for you? Please use a scale from 0 to 10, where 0 = not important at all and 10 = very important.

0	1	2	3	4	5	6	7	8	9	10

Emergency ophthalmology

27.2. Which would you say are the MAIN BARRIERS when initiating treatments for keratitis?

27.2. Specifically, thinking about the barriers when prescribing treatments for keratitis, how important is the cost for you? Please use a scale from 0 to 10, where 0 = not important at all and 10 = very important.

0	1	2	3	4	5	6	7	8	9	10
